# Immunoregulatory role of quercetin in lung cancer

**DOI:** 10.1038/s41420-026-03312-7

**Published:** 2026-09-14

**Authors:** Christine Carvalho, Elvedina Nendel, Susanne Mittler, Sonja Trump, Adriana Geiger, Jonas Willar, Mircea T. Chiriac, Stefan Wirtz, Carol I. Geppert, Susetta Finotto

**Affiliations:** 1 Department of Molecular Pneumology, Friedrich Alexander University Erlangen-Nürnberg (FAU), Universitätsklinikum Erlangen, Erlangen, Germany; 2 Department of Internal Medicine 1, Friedrich Alexander University Erlangen-Nürnberg (FAU), Universitätsklinikum Erlangen, Erlangen, Germany; 3 Institute of Pathology, University Hospital, Friedrich-Alexander-Universität Erlangen-Nürnberg, Erlangen, Germany; 4 Bavarian Cancer Research Center (BZKF), Erlangen, Germany; 5 Comprehensive Cancer Center Erlangen-EMN (CCC ER-EMN), Erlangen, Germany; 6 Deutsches Zentrum für Immuntherapie (DZI), Erlangen, Germany

**Keywords:** Tumour immunology, Cancer

## Abstract

Long-term nourishment with quercetin, a naturally occurring plant flavonoid abundant in citrus fruits, buckwheat, and onions, has been associated with reduced risks of chronic diseases, including cancer. In this study, we evaluated the therapeutic potential and the anti-cancer properties of quercetin in lung cancer using in vitro, in vivo, and ex vivo models. Quercetin induced tumor cell cycle arrest via the p53-p21 axis and suppressed tumor cell migration in both the A549 and H520 human lung cancer cell lines. Moreover, it reduced oxidative stress and downregulated the protein expression of oncogenic markers such as EGFR and PD-L1 on the surface of A549 cells in a dose-response manner. Quercetin significantly attenuated the proliferation and migration of the tumor cells. In an in vivo model, quercetin decreased the tumor load without affecting the body weight. In the ex vivo model, quercetin treatment resulted in the induction of interferon alpha-2 (IFN-α2), a well-known anti-tumor cytokine. These findings demonstrate that quercetin exerts potent anti-tumoral and anti- inflammatory effects in lung cancer by regulating cell cycle, oxidative stress, and immune pathways.

**Figure:**
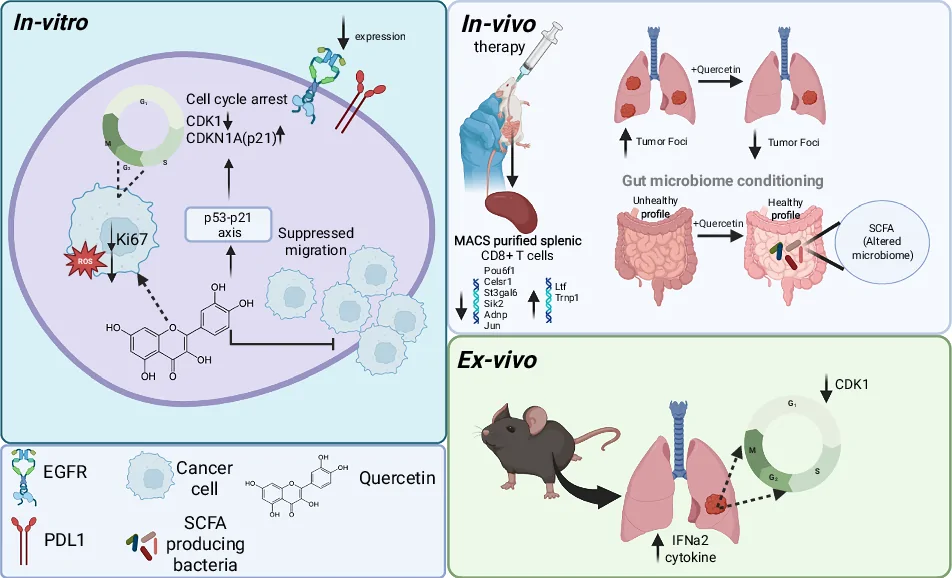
**Overview of quercetin-induced cellular modulation in lung cancer** *[Created in BioRender]* Quercetin had a direct effect on the tumor cell cycle, where it upregulated CDKN1A and downregulated CDK1 in both murine models and human lung cancer cell lines. Quercetin regulated the p53-p21 axis, decreased EGFR, PD-L1, and Ki67 expression, and also showed a strong inhibitory effect on tumor cell migration. It also conditioned the gut microbiome, promoting an increased abundance of SCFA-producing bacteria. At the same time, it led to an increase in IFN-α2 cytokine production. Quercetin also reprogrammed the splenic CD8 + T cell transcriptome, most notably by upregulating the tumor suppressor gene Ltf while also downregulating several oncogenes involved in proliferation and metastasis, namely POU6F1, Celsr1, ST3GAL6, Sik2, Adnp, and Jun.

**Overview of quercetin-induced cellular modulation in lung cancer** *[Created in BioRender]* Quercetin had a direct effect on the tumor cell cycle, where it upregulated CDKN1A and downregulated CDK1 in both murine models and human lung cancer cell lines. Quercetin regulated the p53-p21 axis, decreased EGFR, PD-L1, and Ki67 expression, and also showed a strong inhibitory effect on tumor cell migration. It also conditioned the gut microbiome, promoting an increased abundance of SCFA-producing bacteria. At the same time, it led to an increase in IFN-α2 cytokine production. Quercetin also reprogrammed the splenic CD8 + T cell transcriptome, most notably by upregulating the tumor suppressor gene Ltf while also downregulating several oncogenes involved in proliferation and metastasis, namely POU6F1, Celsr1, ST3GAL6, Sik2, Adnp, and Jun.

## Main

Lung cancer is the second most frequently diagnosed cancer globally, according to the International Agency for Research on Cancer Global Observatory. In 2020, the worldwide estimate of newly diagnosed lung cancer cases rose to approximately 2.2 million, accounting for 11.4% of new cancer cases. Despite this, lung cancer remains the leading cause of cancer-related mortality, with estimated new deaths of 1.8 million (18.0%) [1].

NSCLC is any type of epithelial lung cancer (80%) other than small cell lung cancer (SCLC) (20%) [2].

The most common types of NSCLC include squamous cell carcinoma, large cell carcinoma (SCC), and adenocarcinoma (ADC). Although NSCLCs are associated with cigarette smoke, adenocarcinomas can also be found in patients who have never smoked in their life [3].

Particulate matter (PM) is a complex mixture of tiny solid particles and liquid droplets that are suspended in the air, which vary greatly in size, composition, and origin, and play a major role in air pollution and human health concerns, including lung cancer. These particles are generated from vehicle exhaust, tobacco smoke, and industrial emissions and can travel into cells and organs, posing serious health risks, hence affecting the non-smokers as well [4].

The increasing incidence of NSCLC over the last decade has driven emphasis on cancer research [5]. This has resulted in a deeper understanding of the molecular drivers of lung cancer, such as mutations in the tumor suppressor gene p53, overexpression of Epidermal Growth Factor Receptor (EGFR), and upregulation of programmed death ligand (PD-L1), which often dictate the aggressive phenotype and immune-invasive nature of the disease. In order to improve treatment outcomes, an in-depth understanding of drug resistance mechanisms and the development of combination therapies targeting these specific signaling axes have become essential [6].

Natural biomolecules rich in secondary metabolites contain phyto-mediated content that exhibits a broad spectrum of biological activities, laying the basis for cancer prevention and treatment. The use of natural or synthetic substances to inhibit carcinogenesis is defined as Chemoprevention [7].

Epidemiological studies suggest that long-term consumption of a diet rich in fruits and vegetables reduces the risk of chronic diseases, particularly cancer. Such diets may reduce exposure to harmful substances and limit the activation of procarcinogens while maximizing the intake of beneficial nutrients like isothiocyanates, unsaturated fatty acids, polyphenolic terpenoids (PPT), selenium, terpenes, etc. In 1930, a new substance was isolated from oranges and was found to decrease the capillary permeability and was initially believed to be a member of a new class of vitamins, termed vitamin P. However, it was later identified as a flavonoid (rutin) [8].

Flavonoids are secondary metabolites, characterized by a benzopyrone ring in the core with phenolic or poly-phenolic groups attached at different positions. They comprise a large class of naturally occurring polyphenolic compounds ubiquitously found in the plant kingdom [7].

Flavonoids accumulate in plant cell vacuoles and occur in glycosylated form, which makes them water soluble and renders them chemically less reactive [9]. Flavonoids have a remarkable ability to interact with proteins [8]. The antioxidant activity of flavonoids is closely associated with their molecular structure, particularly the total number and the location of the hydroxyl (–OH) groups. Furthermore, it is also influenced by the conjugation and resonance effects, the surrounding environment, which determines the most thermodynamically favored antioxidant site, and the specific antioxidant mechanism for a compound [7].

Quercetin (QUE; 3,5,7,30, 40 -penta-hydroxy-flavone), an antioxidant flavonoid, is the major representative of the flavonoid subclass of flavanols. Quercetin is widely distributed in fruits and vegetables. According to the US Department of Health and Human Services, the average daily intake of quercetin in humans is approximately 25mg [10]. Onions are particularly rich in quercetin, being 0.03–0.28 mg/100 g of the fresh weight (FW) of white and yellow onions, while red onion varieties exhibit the highest content (around 1.31 mg/100 g FW) [11]. Quercetin exhibits a wide range of biological effects, including antioxidant, anticarcinogenic, anti-inflammatory, anti-diabetic, and antimicrobial activities [12–14]. In plants, quercetin is present in the form of glycosides, which are hydrolysed by Β-glycosidases in the intestine that catalyse the hydrolysis of glycosidic bonds before being absorbed into the enterocytes, where they are metabolized into quercetin conjugates. Low bioavailability of quercetin limits its systemic effects which results in the differences between in-vitro and in-vivo studies [15]. Quercetin elicits biphasic, dose-dependent effects: (1) at low concentrations, it acts as an antioxidant and thus elicits chemopreventive effects; (2) at high concentrations, it functions as a pro-oxidant and elicits chemotherapeutic effects [11]. Flavonoids serve as exogenous antioxidants and neutralize free radicals through four mechanisms, namely: (1) the inhibition of nitric oxide synthase activity, (2) inhibition of xanthine oxidase activity, (3) modulation of channel pathways, or (4) interaction with other enzyme systems. Positive effects on human health have resulted in an increasing effort being made to isolate these compounds from various plants [7].

Limited research has been conducted regarding flavonoids and their impact on NSCLC [7]. Particularly, the orchestration of a systemic immune response via the gut-lung axis and the transcriptomic reprogramming of immune cells represent a significant gap in our understanding of flavonoid-mediated lung cancer inhibition.

Therefore, the objective of this study was to examine how natural compounds like quercetin, found in plants, can mitigate and help suppress lung cancer development.

## Results

### Quercetin modulates the expression of cell cycle and apoptotic regulatory genes in lung tumor cells

**Fig. 1:**
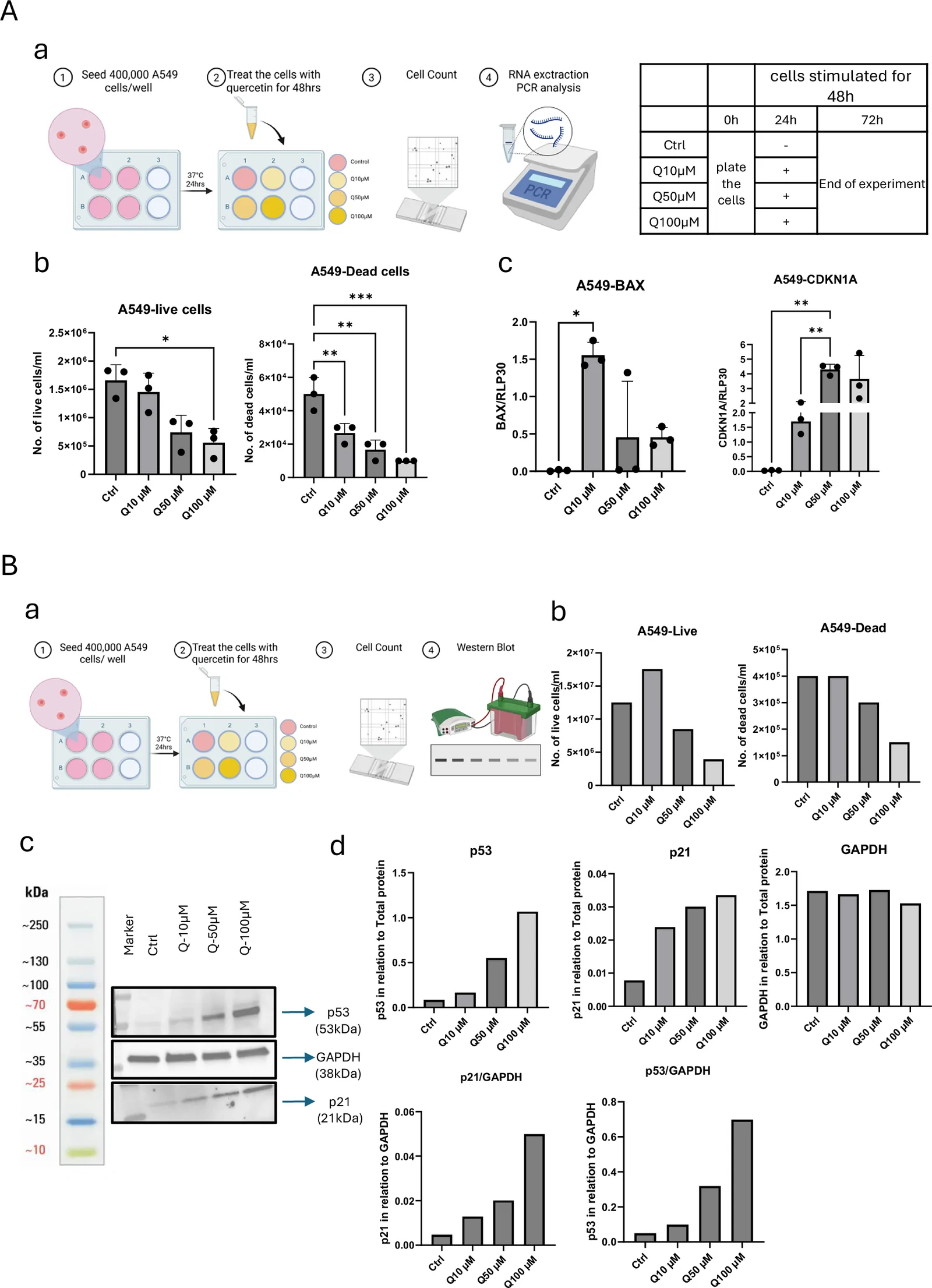
Quercetin triggered p53-p21 mediated cell cycle arrest and upregulated apoptotic genes in lung tumor cells. **A**: Quercetin modulates the expression of cell cycle and apoptotic regulatory genes in lung tumor cells **a** Schematic of the experimental design- 4x10^5^cells were cultured for 24 h, treated as indicated with 10 µM, 50 µM and 100 µM quercetin and harvested for viability and gene expression. **b** Quantification of the live and dead cells was determined using trypan blue exclusion. **c** Relative mRNA expression of BAX and CDKN1A (p21) measured by qPCR and normalized to housekeeping control (RLP30). Data are presented as the mean with SD from *n* = 3 independent experiments. Statistical significance was assessed by the Brown-Forsythe ANOVA test, *p* < 0.05 was considered significant. **B**: Quercetin-induced lung tumor cell cycle arrest is associated with activation of the p53-p21 axis. Quercetin induced cell cycle arrest via p53- dependent pathway: **a** Schematic of the experimental design- 4x10^5^ cells were cultured for 24 h, treated as indicated, and harvested for viability and protein analyses *[Created in BioRender]*. **b** Live and dead cell counts were determined by trypan blue exclusion **c** Representative western blot showing p53, p21, and GAPDH protein levels **d** Quantification of p53 and p21 protein expression relative to total protein and to GAPDH, with GAPDH also normalized to total protein. Data are presented as the mean with SD from *n* = 1 independent experiment.

To characterize the cytostatic and pro-apoptotic effect of quercetin on lung cancer, we first exposed the lung adenocarcinoma cell line A549 to increasing doses of quercetin and analysed cell survival and death (Fig. 1A.a). We observed a significant reduction in viable cell numbers upon administration of 100 µM quercetin. The effect of quercetin on cell death followed the same trend as that of the live cells (Fig. 1A.b). When evaluating apoptotic signaling, we found a significant induction of BAX at the lowest dose of quercetin (Fig. 1A.c). However, the cell cycle inhibitor CDKN1A (also known as asp21) was found to be upregulated by 50 µM quercetin dose, indicating that quercetin induced lung tumor cell cycle arrest (Fig. 1A.c). No significant difference in the CDK1 expression was observed (Supplementary Fig. S1a). A similar trend was observed in the H520 cell line (Supplementary Fig. S2a, b).

### Quercetin induced lung tumor cell cycle arrest via activation of the p53-p21 axis

To investigate whether quercetin modulates tumor cell cycle progression through a p53-dependent pathway, A549 cells were cultured and treated as indicated (Fig. 1B.a). p53 is a critical tumor suppressor that regulates cell cycle arrest, DNA repair, and apoptosis, and its role has been described in previous studies [16]. Quercetin treatment resulted in a decrease in the proportion of live as well as dead tumor cells (Fig. 1B.b). Western blot analysis consistently showed increased expression of p21 and p53 proteins in treated cells, with GAPDH serving as a housekeeping gene (Fig. 1B.c and Supplementary Fig. S3A–E). Quantification revealed that p21 and p53 protein levels increased relative to total protein in a dose-dependent manner (Fig. 1B.d). These findings indicate that quercetin may induce tumor cell cycle arrest through upregulation of p53 and its downstream effector p21, thereby contributing to impaired cell proliferation.

### Comparative analysis of quercetin’s anti-proliferative mechanisms in lung adenocarcinoma and squamous carcinoma cell lines

**Fig. 2:**
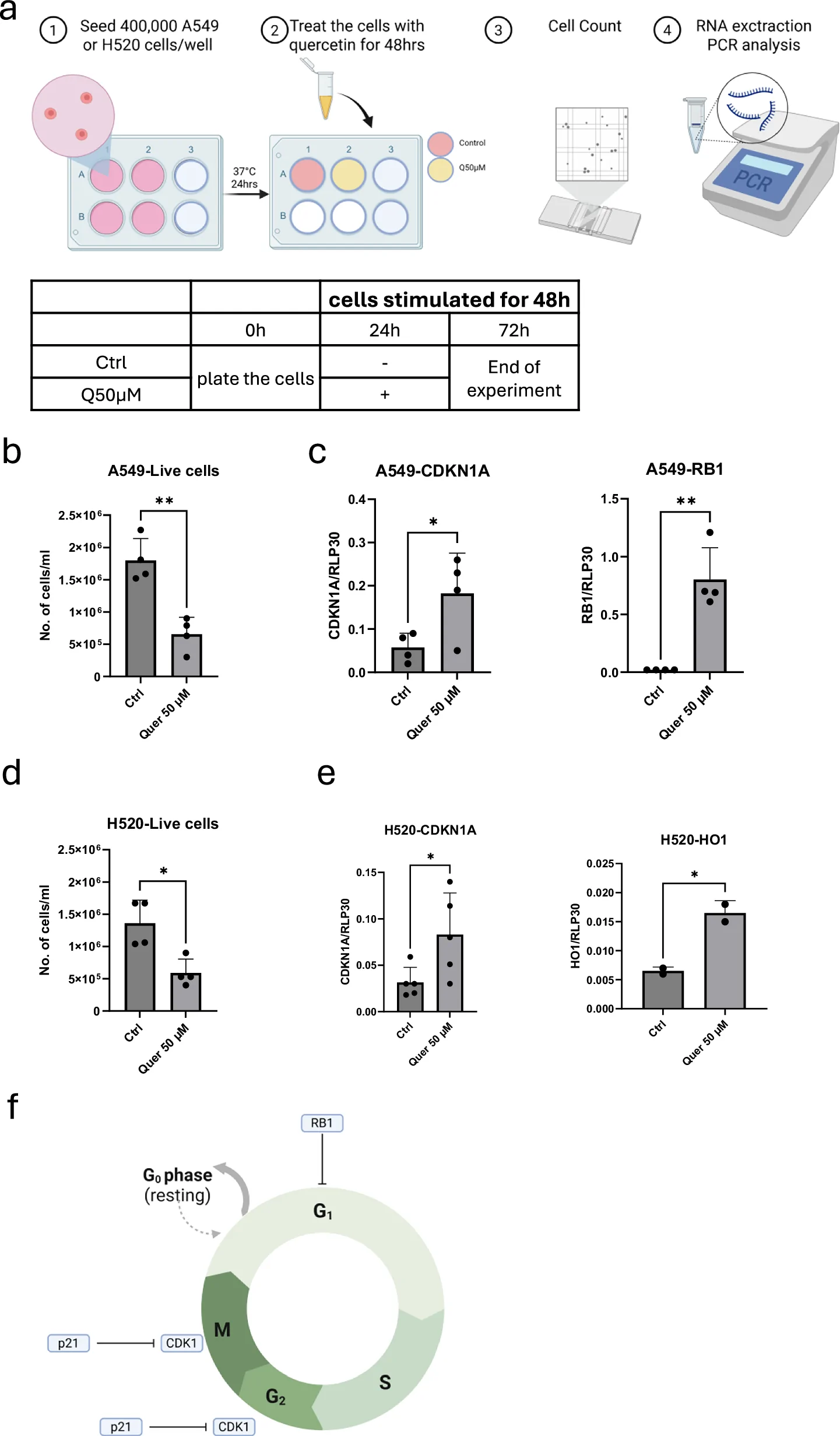
Quercetin induced lung cancer cell cycle arrest via p21. **a** Schematic of the experimental design- 4x10^5^A549 and H520 cells were cultured for 24 h, treated with 50 µM quercetin and harvested for viability and gene expression *[Created in BioRender]*. **b** Quantification of live A549 cells determined using trypan blue exclusion. **c** Relative mRNA expression of CDKN1A (p21) and RB1 measured by qPCR and normalized to the housekeeping control (RLP30). **d** Quantification of the live H520 cells was determined using trypan blue exclusion. **e** Relative mRNA expression of CDKN1A (p21) and HO1 measured by qPCR and normalized to housekeeping control (RLP30). **f** Illustration of the cell cycle phases (G1, S, G2, M, G0) with p21 and RB1 inhibiting cell cycle progression and promoting arrest under stress or checkpoint activation. Data are presented as the mean with SD from *n* = 3 independent experiments. Statistical significance was assessed by an unpaired t-test. The significance threshold was determined according to specified limits: * (one asterisk): *p* < 0.05 (indicates a statistically significant difference) ** (two asterisks): *p* < 0.01 (indicates a highly significant difference) *** (three asterisks): *p* < 0.001 (indicates a very highly significant difference) ns (not significant): *p* > 0.05 (indicates no statistically significant difference).

A549 and H520 cells were exposed to 50 µM of quercetin for 48 h (Fig. 2a). Quercetin treatment led to a significant decrease in the number of live A549 cells (Fig. 2b). At the molecular level, a significant upregulation of CDKN1A was observed in the A549 cells (Fig. 2c). A similar trend was seen in the H520 cells (Figs. 2d, e), indicating an anti-proliferative effect in both lung cancer subtypes, consistent with its role in cell cycle arrest (Fig. 2f). In addition, a significant increase in RB1 expression was detected specifically in A549 cells (Fig. 2c), supporting regulation of the G1 to S phase transition in this adenocarcinoma model. In the H520 cells, expression of the HO-1 gene was significantly upregulated, suggesting the anti-oxidative role of quercetin in neutralizing oxidative stress (Fig. 2e). By contrast, no significant changes were observed in the number of dead cells and in the expression of CDK1, BAX, HO-1, and NQO1 genes in the A549 cell line (Supplementary Fig. S4a–c). Similarly, no significant changes were observed in the number of dead cells and in the expression of CDK1, RB1, BAX, and NQO1 genes in the H520 cell line (Supplementary Fig. S5a–c). Taken together, these results suggest that quercetin primarily exerts its anti-proliferative effect through induction of cell cycle arrest via the p53–p21–RB1 pathway rather than apoptosis.

### Quercetin induced a dose-dependent reduction of EGFR and PD-L1

**Fig. 3:**
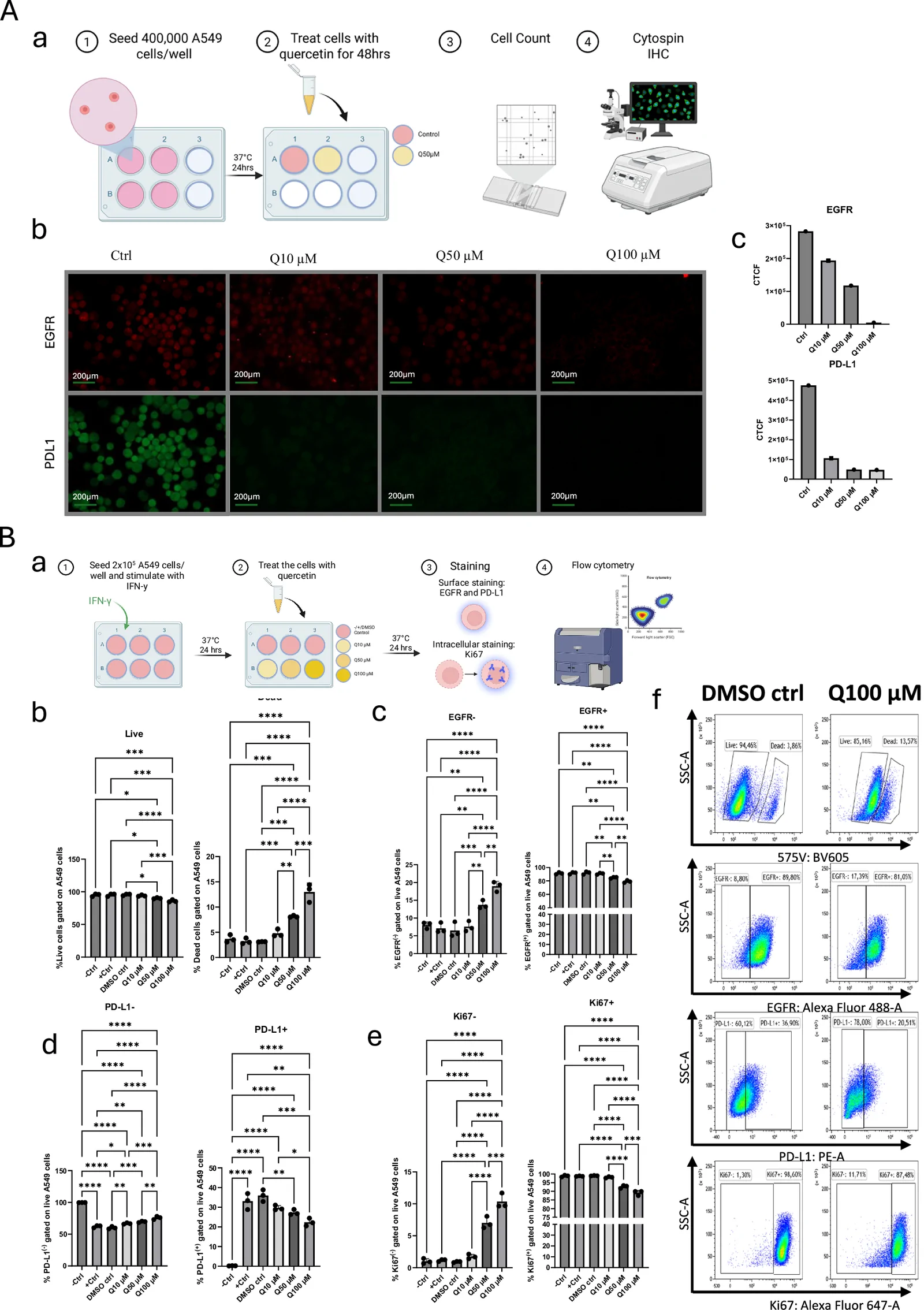
Quercetin affected the viability and, downregulated EGFR and PD-L1 expression of A549 cells. **A**: Quercetin decreased surface expression of EGFR and PD-L1 in lung cancer cells. **a** Experimental design of dose-response experiment using cytospin *[Created in BioRender]*. **b** Representative IHC images of A549 cells stained for EGFR and PD-L1 post- treatment with quercetin at the indicated concentrations **c** Quantification of CTCF for EGFR and PD-L1: data were calculated across 1 random field, evaluating five individual cells per field at 40X magnification (*n* = 1). Data are presented as the mean with SD. Statistical significance was assessed by Ordinary one-way ANOVA. The significance threshold was determined according to specified limits: * (one asterisk): *p* < 0.05 (indicates a statistically significant difference) ** (two asterisks): *p* < 0.01 (indicates a highly significant difference) *** (three asterisks): *p* < 0.001 (indicates a very highly significant difference) ns (not significant): *p* > 0.05 (indicates no statistically significant difference). **B**: Quercetin attenuates tumor phenotype and proliferation **a** Schematic of the experimental design – A549 cells were seeded at a density of 2 x 10^5^ cells per well, stimulated with 50 ng/ml IFN-gamma and treated with different doses of quercetin (10 µM, 50 µM, 100 µM) for 24 h and further processed for flow cytometric analysis. **b** Quantitative flow cytometric analysis showing the percentage of live and dead cells gated on the total A549 cells following treatment with quercetin. **c** Quantitative flow cytometric analysis showing the percentage of EGFR^+^ and EGFR^-^ cells gated on the live A549 cells following treatment with quercetin. **d** Quantitative flow cytometric analysis showing the percentage of PD-L1^+^ and PD-L1^-^ cells gated on the live A549 cells following treatment with quercetin. **e** Quantitative flow cytometric analysis showing the percentage of Ki67^+^ and Ki67^-^ cells gated on the live A549 cells following treatment with quercetin. **f** Representative flow cytometry dot plots showing live/dead; EGFR; PD-L1 and Ki67 (+) and (–) shifts in A549 cells treated with DMSO vehicle control and 100 µM quercetin. Data are presented as the mean with SD from *n* = 3 independent experiments. Statistical significance was assessed by the ordinary one-way ANOVA test; *p* < 0.05 was considered significant. The significance threshold was determined according to specified limits: * (one asterisk): *p* < 0.05 (indicates a statistically significant difference) ** (two asterisks): *p* < 0.01 (indicates a highly significant difference) *** (three asterisks): *p* < 0.001 (indicates a very highly significant difference) ns (not significant): *p* > 0.05 (indicates no statistically significant difference).

To evaluate the effect of quercetin on the expression of EGFR and PD-L1, A549 cells were subjected to cyto-spin preparation followed by immunofluorescence staining for EGFR and PD-L1 (Fig. 3A.a). Representative images revealed a dose-dependent decrease in EGFR (red) and PD-L1 (green) fluorescence intensity (Fig. 3A.b). To quantify target protein expression, CTCF of EGFR and PD-L1 was calculated across five random fields, evaluating five individual cells per field at 40X magnification (*n* = 1). The resulting quantification revealed that quercetin downregulates EGFR and PD-L1 expression in A549 cells in a dose-dependent manner. (Fig. 3A.c).

### Dose-dependent effects of quercetin on phenotypic markers and cell viability of A549 cells

To evaluate the phenotypic and proliferative changes induced by quercetin, flow cytometric analysis was performed on A549 cells to monitor the expression of EGFR, PD-L1, and Ki67 (*n* = 3) (Fig. 3B.a). Cell viability analysis via flow cytometry revealed that quercetin treatment led to a simultaneous increase in live A549 cells and a corresponding increase in the dead cell population (Fig. 3B.b). Flow cytometry data demonstrated that quercetin affected the expression of EGFR, PD-L1, and Ki67 in a dose-dependent manner. This effect was visually characterized by a progressive leftward shift (negative population) of the fluorescent cell populations on the flow cytometry histograms. This leftward shift towards the negative population indicates that treatment with increasing doses of quercetin effectively downregulates the surface expression of EGFR (Fig. 3B.c) and PD-L1 (Fig. 3B.d), and intracellular expression of Ki67 (Fig. 3B.e) on A549 cells. The gating strategy used is shown in Supplementary Fig. 6

### Effect of quercetin on oxidative stress

**Fig. 4:**
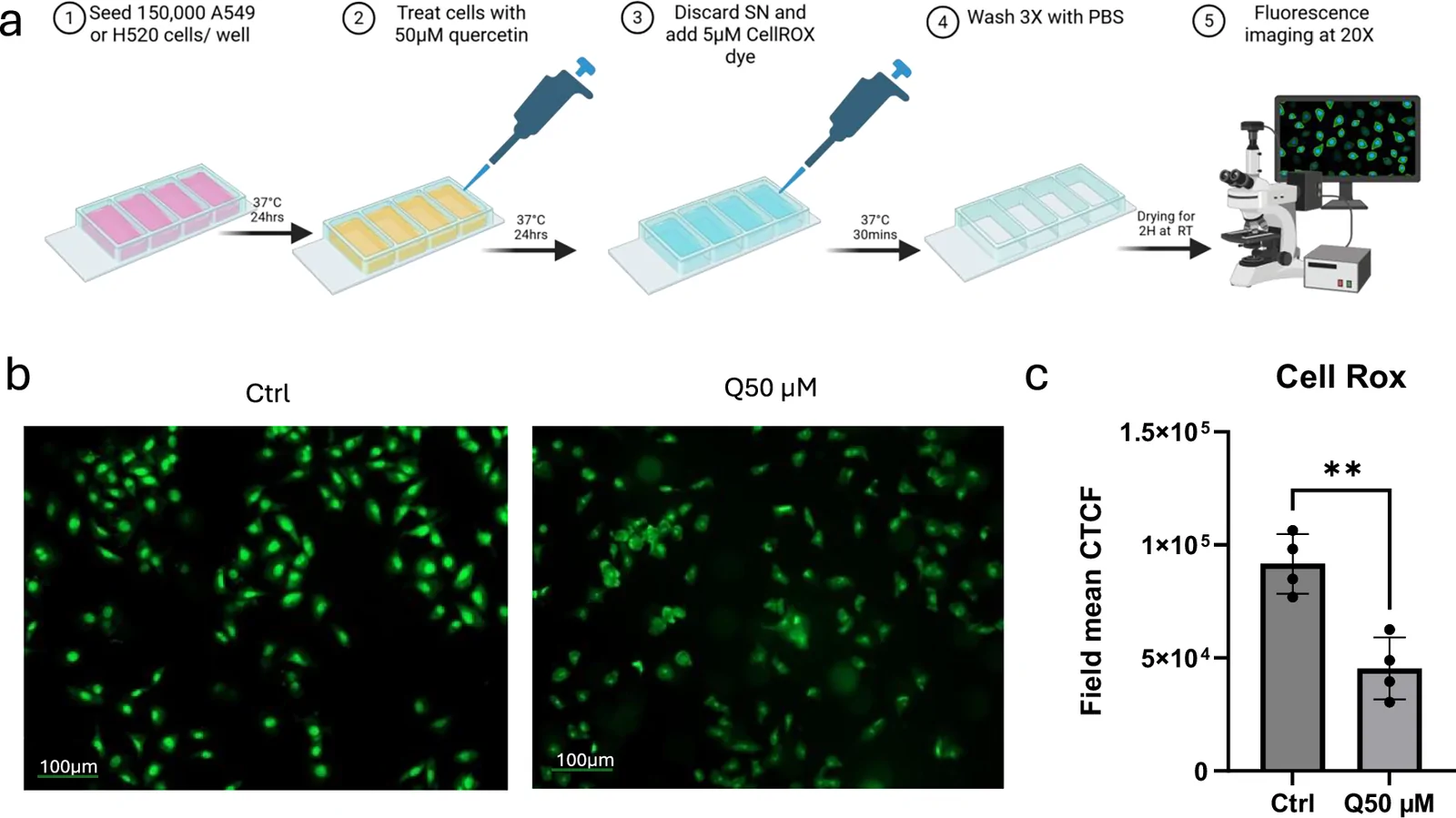
Quercetin reduced oxidative stress in the A549 cells. **a** Experimental design: A549 cells were treated with 50 µM of quercetin for 48 h and later stained with Cell ROX reagent to detect ROS *[Created in BioRender]*. **b** Representative fluorescence microscopy images showing ROS in bright green. **c** Quantification of CTCF: Data were calculated across 4 random fields, evaluating 5 individual cells per field at 40X magnification. Statistical significance was assessed by the ordinary one-way ANOVA test. Data are presented as the mean with SD. Statistical significance was assessed by a paired t-test. The significance threshold was determined according to specified limits: * (one asterisk): *p* < 0.05 (indicates a statistically significant difference) ** (two asterisks): *p* < 0.01 (indicates a highly significant difference) *** (three asterisks): *p* < 0.001 (indicates a very highly significant difference) ns (not significant): *p* > 0.05 (indicates no statistically significant difference).

Quercetin treatment led to a reduction in oxidative stress in A549 cells, as shown by decreased green fluorescence signal following Cell-ROX staining (Fig. 4a, b). For quantification, images were captured at 20X magnification from four random fields per condition. Quantification of CTCF in five individual cells per field confirmed this observation, demonstrating a decrease in intracellular ROS levels following 48h  quercetin treatment (Fig. 4c). Quercetin treatment did not affect the oxidative stress (green signal) in H520 cells (Supplementary Fig. S7a, b).

### Quercetin exhibited an anti-migratory effect on lung tumor cells

**Fig. 5:**
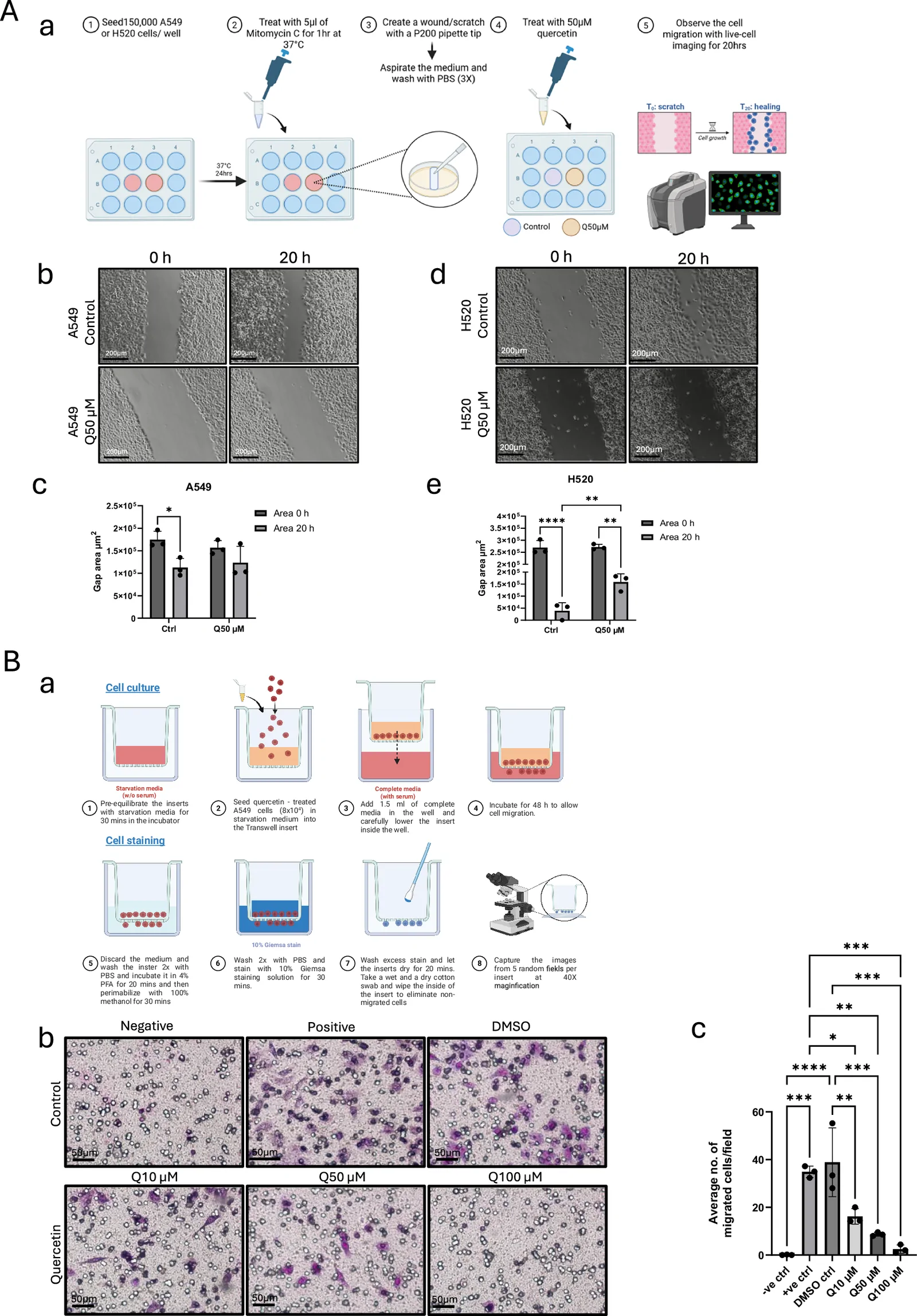
Quercetin impaired cellular motility and migration in lung tumor A549 cells. **A**: Quercetin inhibited the migration of A549 and H520 cells in a wound healing assay. **a** Schematic representation of scratch assay. **b** Representative time-lapse microscopy images of A549 monolayers at 0 h and 24 h time point under control and quercetin (50 µM) treated conditions *[Created in BioRender]*. **c** Quantification of the wound gap area over time, expressed as Gap area µm [2] in A549 cells (*n* = 4). **d** Representative time-lapse microscopy images of H520 monolayers at 0 h and 2 h time points under control and quercetin (50 µM) treated conditions. **e** Quantification of the wound gap area over time, expressed as Gap area µm [2] in H520 cells (*n* = 3). Data are presented as mean with SD. Statistical significance was assessed by 2way ANOVA. The significance threshold was determined according to specified limits: * (one asterisk): *p* < 0.05 (indicates a statistically significant difference) ** (two asterisks): *p* < 0.01 (indicates a highly significant difference) *** (three asterisks): *p* < 0.001 (indicates a very highly significant difference) ns (not significant): *p* > 0.05 (indicates no statistically significant difference). **5B**: Migratory capacity of A549 cells was inhibited by quercetin **a** Schematic of the experimental design – A549 cells were seeded at a density of 8 × 10^4^ cells per trans-well insert and incubated for 48 h to allow migration. Migrated cells were subsequently fixed and stained with 10% Giemsa solution. Images were captured and quantified across 5 random fields at 40X magnification under an inverted microscope *[Created in BioRender]*. **b** Representative microscopic images of Giemsa-stained A549 cells post 48 h transwell migration assay under control and quercetin-treated conditions. **c** Quantification of migrated cells on the lower membrane surface at 48 h, calculated as the mean cell count from 5 random fields per insert. Data are presented as mean with SD from *n* = 3 independent experiments. Statistical significance was assessed by the ordinary one-way ANOVA test. The significance threshold was determined according to specified limits: * (one asterisk): *p* < 0.05 (indicates a statistically significant difference) ** (two asterisks): *p* < 0.01 (indicates a highly significant difference) *** (three asterisks): *p* < 0.001 (indicates a very highly significant difference) ns (not significant): *p* > 0.05 (indicates no statistically significant difference).

To assess the effect of quercetin on tumor cell migration, a wound healing assay was performed in the A549 (*n* = 3) and H520 (*n* = 3) cell lines (Fig. 5a). Representative time lapse images acquired at 0 h and 20 h show a decrease in the A549 cell migration with quercetin treatment (50 µM), while in the control condition the cells migrated and led to the closure of the wound gap (Fig. 5b). Quantitative analysis confirmed that quercetin treatment significantly inhibited the gap closure (Fig. 5c), indicating that quercetin decreased A549 cell migration. Similar results were obtained with the H520 cell line (Figs. 5d, e). The time-lapse images were converted into videos (960 x 720 pixels) using the BZ-X800 series software and are provided as Supplementary videos S1a, b, c - 4a, b, c. An example illustrating the outlining method used for gap area measurement using ImageJ is provided (Supplementary Fig. S8a, b).

### Transwell migration assay of A549 cells following treatment with quercetin

To further confirm the functional impact of quercetin on the migration potential of the A549 cells, a trans-well assay was performed (*n* = 3) (Fig. 5B.a). Representative microscopic images revealed a striking dose-response reduction in the density of migrated cells, characterized by visibly fewer stained blue-violet cells in the quercetin-treated groups compared to the crowded vehicle control (Fig. 5B.b). Subsequent quantitative analysis confirmed the statistical significance of quercetin treatment (Fig. 5B.c). An example illustrating the outlining method used to quantify the number of migrated cells using ImageJ is provided (Supplementary Fig. S8c).

### Quercetin reduced lung tumor without affecting inflammation and body weight

**Fig. 6:**
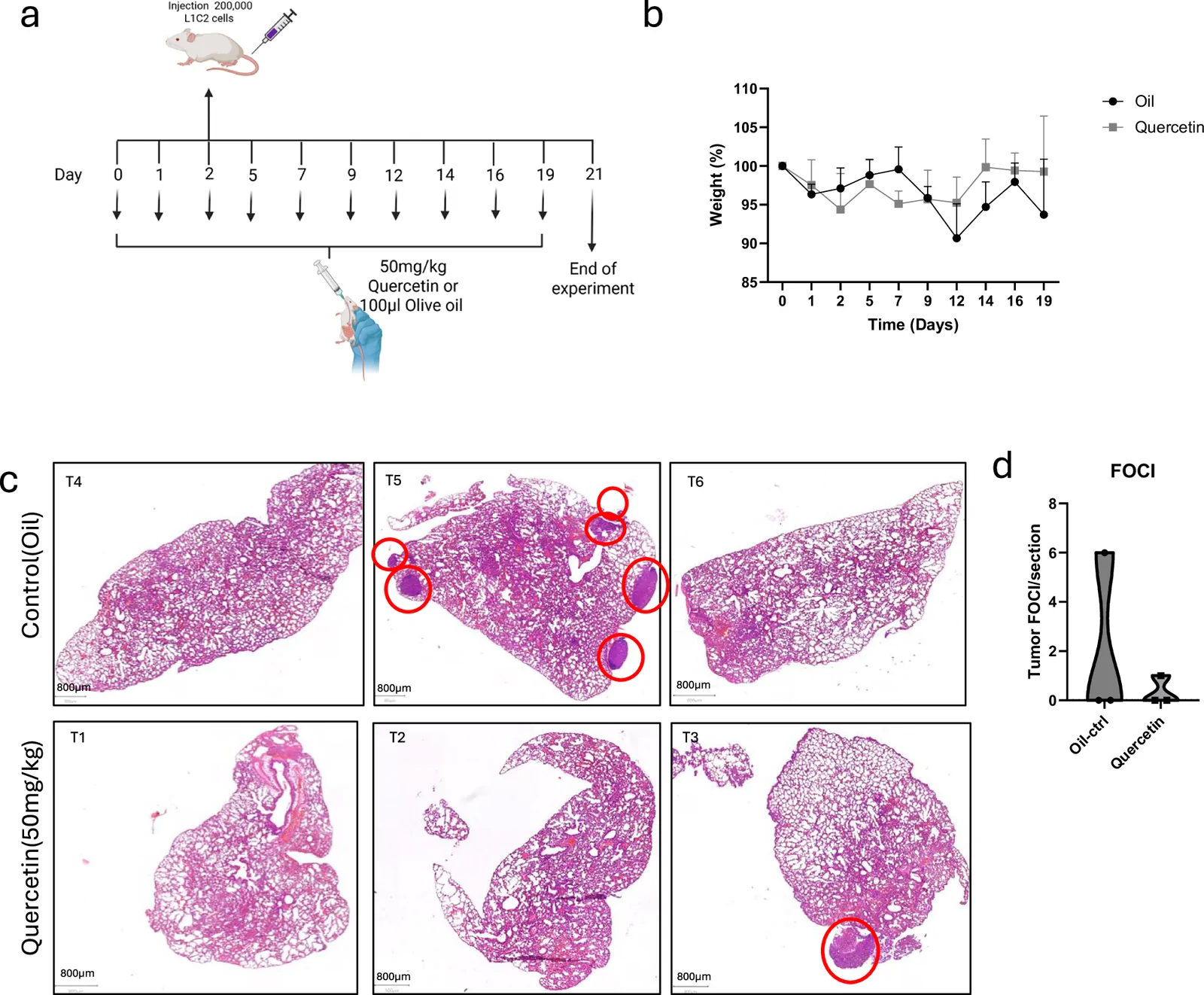
Effects of quercetin treatment in the murine model of lung cancer. **a** BALB/c mice were injected with 2×10^5^ L1C2 tumor cells on day 0 to induce lung tumor and treated with quercetin (50 mg/kg/day, oral gavage) or olive oil according to the experimental scheme** b** Body weight was monitored throughout the study *[Created in BioRender]* **c** Representative H&E staining of lung tissue from control and quercetin-treated mice is shown to illustrate histopathological differences **d** Tumor burden was evaluated by measuring the number of tumor foci (red circled) in the lung section. A violin plot was generated to visualize differences in tumor foci between experimental groups. Data are represented as percentages of total sequences in each group. Statistical significance was assessed by Welch’s t-test, *p* < 0.05 was considered significant.

To evaluate the potential anti-tumoral effect of quercetin, BALB/c mice were injected with 2 × 10^5^ L1C2 cells in the tail vein on day 2 to establish an experimental lung metastasis model, focusing primarily on the colonization phase of tumor progression. Quercetin was delivered to the mice via oral gavage according to the experimental scheme (Fig. 6a). Body weight of the mice was monitored throughout the study and it revealed that the quercetin did not affect the weight of the mice, suggesting that the treatment was well tolerated and did not adversely affect the health status of the mice (Fig. 6b). Representative lung microscopic images and tumor burden analysis showed detectable tumor foci in the lung sections of both the groups (Fig. 6c). In the control group, a total of 6 foci per lung section were observed, whereas in the quercetin group, only 1 tumor foci per lung section was detected (Fig. 6d). Preliminary results might suggest that quercetin administration is associated with a marked reduction in lung tumor occurrence. No significant difference was observed in the overall lung tumor load (Supplementary Fig. S9a) and lung inflammation (Supplementary Fig. S9b) across the 2 groups.

### Quercetin modulated gut microbiome towards beneficial SCFA-producing bacteria

We next reasoned that quercetin could influence the gut microbiome. Thus, we analysed the stool samples from the mice and found that quercetin treatment altered the gut microbiome by shifting its composition towards beneficial SCFA-producing taxa, specifically at the genus level for *Butyribacter*, *Alistipes*, *Lachnospiraceae* and *Odoribacter*, suggesting that quercetin may promote a healthier microbial profile (Supplementary Fig. S10a–e).

### Quercetin reprogrammed the splenic CD8+T cell transcriptome towards an anti-tumor phenotype

To evaluate the efficacy of quercetin (solubilized in olive oil) in comparison to an untreated control mice (no olive oil treatment), BALB/c mice were injected with 5 × 10^5^ L1C2 cells in the tail vein on day 0 to establish an experimental lung metastasis model, focusing primarily on the colonization phase of tumor progression. Quercetin supplementation significantly reprogrammed the splenic CD8 + T cell transcriptome, most notably by upregulating the tumor suppressor gene Ltf (Supplementary Fig. S11a, b). Quercetin treatment significantly downregulated several oncogenes involved in proliferation and metastasis, namely POU6F1, Celsr1, ST3GAL6, Sik2, Adnp, and Jun (Supplementary Table S5).

### Ex vivo analysis showed altered gene expression and reduced inflammation with quercetin treatment in a lung tumor model

**Fig. 7:**
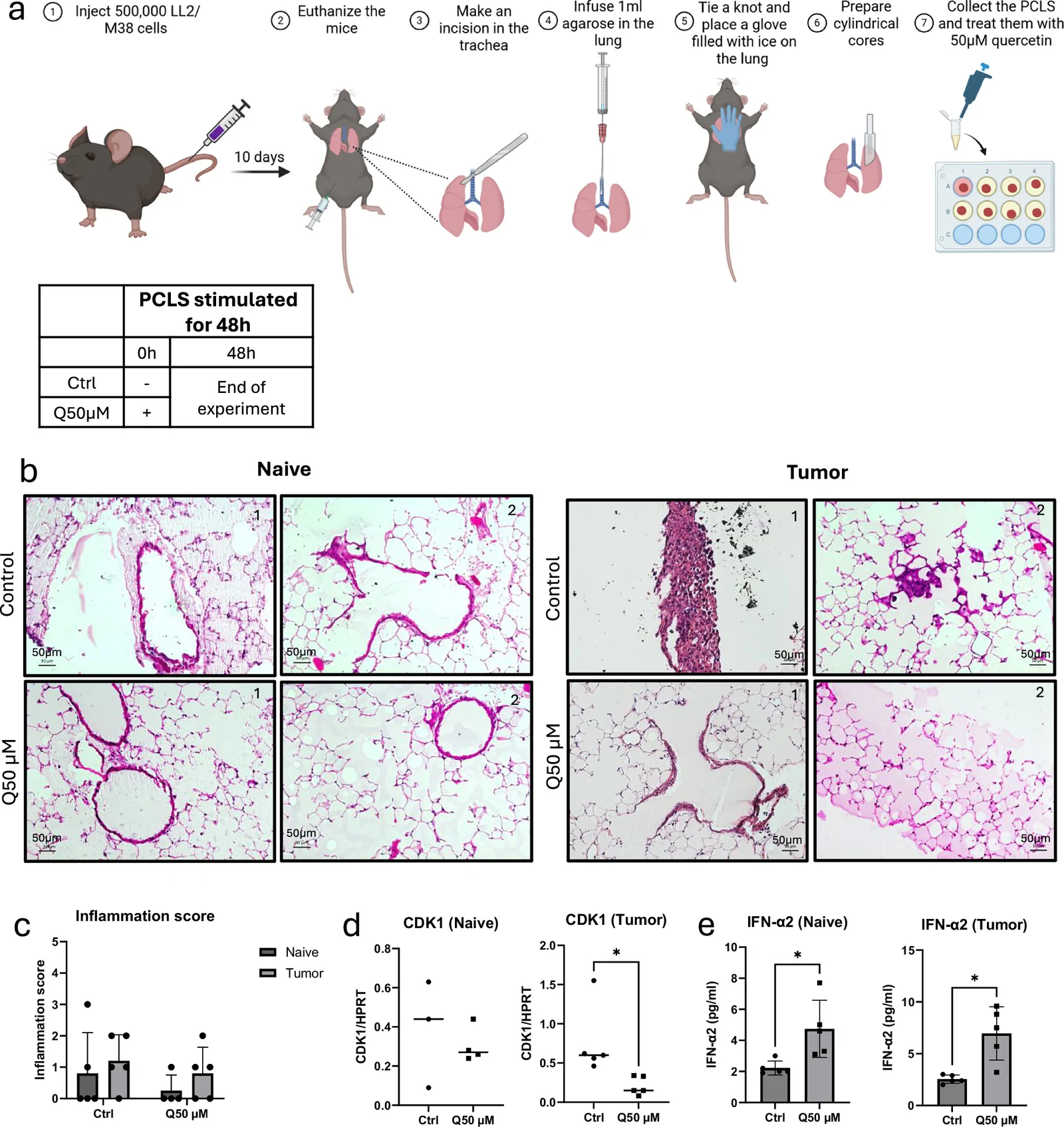
Ex vivo analysis of quercetin treatment via PCLS from tumor-bearing mice. **a** Schematic representation of experimental design: C57BL/6 mice were injected with 5 × 10^5^ LL2/M38 tumor cells, and tumor was allowed to develop for 10 days. At the experimental endpoint, PCLS were prepared from excised lungs and treated ex vivo with quercetin for 48 h *[Created in BioRender]*. **b** Representative H&E–stained lung sections of wild-type and tumor-bearing mice with and without quercetin (50 µM) treatment are shown. **c** Lung inflammation was evaluated using semi-quantitative scoring system and the degree of inflammation was graded on a scale from 1 to 5 (2way ANOVA test) **d** PCLS were further processed for RNA extraction, and the expression cell cycle–regulatory CDK1 gene was quantified **e** Supernatants were analysed using murine inflammation multiplex ELISA according to the manufacturer’s instructions, and the cytokine level of IFN-α2 was measured. Statistical significance was assessed by a paired t-test. The significance threshold was determined according to specified limits: * (one asterisk): *p* < 0.05 (indicates a statistically significant difference) ** (two asterisks): *p* < 0.01 (indicates a highly significant difference) *** (three asterisks): *p* < 0.001 (indicates a very highly significant difference) ns (not significant): *p* > 0.05 (indicates no statistically significant difference).

C57BL/6 mice were injected with 5 × 10^5^ LL2/M38 tumor cells in the tail vein to establish an experimental lung metastasis model, and the tumor was allowed to grow over a period of 10 days. To reduce animal use, precision-cut lung sections (PCLS) were generated at the experimental end-point to obtain numerous lung slices from the same region of the lung. PCLS were cultured with and without quercetin for 48 h (Fig. 7a). Histological assessment (Fig. 7b) of lung inflammation of PCLS sections, showed no effect on inflammation with quercetin treatment (Fig. 7c). Furthermore, tumor cells were visible in the control group but absent in the quercetin treated PCLS.

At molecular level, CDK1 mRNA expression was significantly decreased in quercetin treated PCLS derived from tumor bearing mice (Fig. 7d). While an increase in expression of CDKN1A, BAX and NQO1were noted following quercetin treatment in PCLS derived from tumor bearing mice, these findings did not reach a statistical significance (Supplementary Fig. S12a, b). Similarly, a non-significant pattern of expression of these genes in the naive mice was observed (Supplementary Fig. S12c, d). It is important to note that tumor presence affected the expression of the protective genes like CDKN1A, NQO1, and BAX in comparison to the naive samples (Supplementary Fig. S12). Together, the data demonstrates that quercetin does not induce lung inflammation and modestly affects cell cycle arrest genes in tumor, while highlighting tumor’s suppressive effect on the stress and apoptosis related genes.

### Quercetin enhanced the production of IFN-α2 in the lungs of mice bearing tumors

We next analysed the cytokine levels in the PCLS supernatant using multiplex ELISA. Treatment with quercetin significantly increased IFN-α2 levels in the supernatant of cultured PCLS of tumor-bearing mice (Fig. 7e). The gating strategy used for bead population identification and cytokine quantification is provided in Supplementary Fig. S13a. By contrast, other pro-inflammatory cytokines such as TNF-α, MCP-1, IL-6, IL-1b and IFN-y remain unchanged across both tumor and naive, control vs quercetin treatment groups (Supplementary Fig. S13b–f).

### Overview of quercetin-induced cellular and microbiome modulation in lung cancer

We found that quercetin upregulated the CDKN1A gene and downregulated the CDK1 gene in both murine models and human lung cancer cell lines, indicating a direct effect on tumor cell cycle arrest. Quercetin regulated the p53-p21 axis, decreased EGFR and PD-L1 expression, and also showed a strong inhibitory effect on the migration of A549 cells. It also led to an increase in IFN-α2 cytokine production in the PCLS supernatant of tumor-bearing mice. When the mice were supplemented with quercetin (in vivo*)*, it conditioned their gut microbiome in such a way that it increased the abundance of SCFA-producing bacteria. A deep dive into the splenic CD8 + T cell transcriptome revealed that quercetin induced transcriptomic reprogramming, most notably by upregulating the tumor suppressor gene Ltf while simultaneously downregulating several oncogenes involved in proliferation and metastasis, namely POU6F1, Celsr1, ST3GAL6, Sik2, Adnp, and Jun (Graphical Abstract).

## Discussion

Lung cancer remains one of the leading causes of cancer-related mortality worldwide, and despite advances in targeted and immunotherapeutic strategies, treatment efficacy continues to be limited by drug resistance, metastasis, and toxicity [1]. The cause of lung cancer can also be partially prevented through intervention aiming at reducing environmental oxidative stress components like cigarette smoke, as highlighted in the introduction [4]. Thus, naturally occurring compounds such as quercetin, a dietary flavonoid abundant in fruits and vegetables, have gained attention due to their reported antioxidant and anti-inflammatory properties. However, its clinical application is challenged by poor solubility, low oral bioavailability, and rapid metabolism, which limit its therapeutic concentration in target tissues.

In the present study, we investigated the anti-tumoral properties and underlying molecular mechanisms of quercetin in lung cancer models, including in vitro A549 and H520 cell lines and in vivo murine tumor models. Our results demonstrate that quercetin exerts a strong anti-proliferative effect by inducing cell cycle arrest through activation of the p53–p21–RB1 axis, while simultaneously reducing oxidative stress, suppressing oncogenic signaling molecules: EGFR and PD-L1, and inhibiting tumor cell migration. These findings highlight quercetin’s potential as a multi-targeted compound capable of attenuating tumor progression.

Consistent with the previous research linking quercetin with p53 activation [17], our data shows that quercetin significantly upregulated p53 and its downstream effector p21, leading to impaired cell cycle progression, which was further confirmed by the decrease in live and dead A549 cells. The concomitant increase in RB1 gene expression in A549 cells supports the involvement of a p53-p21-RB1 regulatory axis, which functions to inhibit cyclin-dependent kinases and enforce G1/S phase arrest. Importantly, while apoptosis-associated markers such as BAX showed minor induction at lower quercetin doses, the absence of significant changes in CDK1 gene expression suggests that quercetin primarily mediates cytostasis rather than apoptosis under the conditions tested. This lines up with the theory that quercetin inhibits tumor cell growth predominantly through cell cycle arrest rather than cytotoxicity.

The surface expression of EGFR and PD-L1 decreased following the quercetin treatment. It is known that EGFR overexpression is a hallmark of lung cancer and is associated with enhanced proliferation [18], while PD-L1 contributes to immune evasion [19]. The downregulation of Ki67, a nuclear marker for active proliferation, confirms that quercetin halts cell cycle progression, preventing the rapid proliferation of A549 cells. The dose-dependent suppression of these molecules indicates that quercetin may simultaneously inhibit proliferative and immune escape pathways, reinforcing its potential as a dual-action cancer agent. At the molecular level, quercetin’s ability to reduce the intracellular oxidative stress in our Cell-ROX assay highlights its well-known antioxidant activity, which may contribute to the stabilization of cellular redox balance and the prevention of ROS-driven tumorigenic signaling.

In a functional assay such as a wound healing assay, quercetin noticeably reduced the migratory potential of A549 and H520 cells. The assay revealed that quercetin-treated cells failed to close the wound gap, in contrast to the untreated controls. These findings are consistent with reports showing that quercetin ameliorates the epithelial-mesenchymal transition [20], critical for cancer cell migration and metastasis. Given that cell migration is a rate-limiting step in tumor invasion and metastasis, the progressive reduction in the mobility of A549 cells with treatment with quercetin observed in the transwell assay suggests that quercetin may interfere with key cytoskeletal dynamics in lung adenocarcinoma.

The bioavailability of flavonoids is low, and it has been studied in detail because of their potential health benefits [21, 22]. The therapeutic potential of quercetin was further confirmed by our in vivo results. In murine models, oral administration of quercetin significantly reduced the number of visible tumor foci in lung tissues without affecting body weight. Noticeably, the tumor was located at one specific point in the tissue section compared to the control group, further supporting the anti-migratory behaviour of quercetin. Furthermore, quercetin increased the abundance of SCFA-producing bacteria in the gut of mice bearing lung tumor. Accumulating evidence demonstrates that quercetin modulates gut microbiome composition, specifically enriching known populations of SCFA-producing bacteria [23, 24]. The role of SCFAs in maintaining gut homeostasis has been described well in this study [25]. We recently reported that SCFA induced tumor cell cycle arrest. This study highlights the potential of sodium butyrate (SB) in inhibiting the development of lung cancer by triggering apoptosis, inducing cell cycle arrest, and modulating immune responses. SB activates peripheral blood CD4 + T cells and selectively induces IFN-γ-R1 expression in NK cells in peripheral blood, while inhibiting peripheral blood CD8 + T cells and NK cells. Thus, understanding the mechanisms of action of SB produced by gut bacteria through flavonoid metabolism and how this influences the lung tumor micro-environment and immune system would provide a valuable insight into potentially considering quercetin as a candidate for adjunctive therapies in NSCLC [26].

Quercetin supplementation significantly reprogrammed the splenic CD8 + T cell transcriptome, most notably by upregulating the tumor suppressor gene Ltf, which has an influence on cell proliferation, migration, and invasiveness by inhibiting lipid metabolism [27]. Quercetin significantly downregulated several oncogenes involved in proliferation and metastasis, namely: POU6F1, Celsr1, ST3GAL6, Sik2, Adnp and Jun (Supplementary Fig. S11a, b).

Furthermore, ex vivo analysis of PCLS derived from the tumor-bearing mice revealed upregulation of CDKN1A, BAX, and NQO1 genes, and a significant decrease in CDK1 gene expression. Moreover, quercetin enhanced IFN-α2 production, suggesting a possible immuoe-stimulatory role that may contribute to tumor suppression. Reports also demonstrated that IFN-α serves as both an activator and a regulator of NK function [28].

Collectively, our research identifies quercetin as a multifaceted anti-neoplastic agent that induces cell cycle arrest, decreases tumor cell proliferation, reduces oxidative stress, suppresses tumor cell migration, and downregulates oncogenic markers. Further research should focus on uncovering the upstream regulators of quercetin-induced p53 activation and elucidating oxidative stress pathway genes to pinpoint the specific targets whose expression changes in response to quercetin. Moreover, quercetin also had an influence on the gut microbiome. The limiting factor of this study is that the in vivo experiments were conducted exclusively in female mice, and only 1 mouse per group developed a tumor. To overcome these constraints, future studies should utilize orthotopic lung tumor models to more accurately replicate the primary tumor microenvironment. Additionally, incorporating non-invasive optical imaging such as In vivo Imaging system (IVIS), will be crucial to quantify and track bioluminescent tumor progression in real time. A notable limitation of the current study is that the gut microbiome data is restricted to 16 s rRNA gene sequencing, which characterizes the microbial composition rather than direct metabolic activity. Since additional in vivo and ex vivo metabolomic profiling on the stool sample was not performed, the specific functional metabolite shifts associated with quercetin treatment remain unclear. Future investigations should incorporate targeted metabolomics to validate these compositional shifts.

In summary, quercetin unfolds as a promising candidate to contribute to fighting lung cancer, exerting antioxidant, anti-proliferative, and immunomodulatory effects that collectively obstruct tumor progression.

## Methods

### Cell lines

A549 and H520 cell lines were obtained from the ATCC biobank (Washington, DC, USA) and were stored at –150 °C until use. These cells were thawed, cultured, and prepared for experiments according to standard cell culture protocols. The A549 cell line originates from human lung adenocarcinoma, making it a widely used model for studying non-small cell lung cancer (NSCLC). The H520 cell line, derived from human lung squamous cell carcinoma, is frequently used in research to investigate this form of cancer. The murine LL/2-luc-M38 (LL/2) cell line was purchased from and authenticated by Caliper Life Sciences. All Caliper Life Sciences cell lines were confirmed to be pathogen-free by IMPACT profile I (PCR) at the University of Missouri Research Animal Diagnostic and Investigative Laboratory. L1C2 cells were kindly provided to us by Prof. Dr. med. Rainer Wiewrodt (Pneumology Department at the University Hospital of Münster, Germany). These cell lines were previously described [29]. The A549 and H520 cells were cultured in RPMI 1640 medium (AC-LM-0060; Anprotec Bruckberg, Bavaria Germany) supplemented with 10% FCS (Fetal Calf Serum); 1% P/S(Cat#AC-AB-0024) and 1% L-Glutamine (Cat#AC-AS-0001). Trypsin-EDTA 1X (AC-EZ-0009, Anprotec, Bruckberg, Bavaria, Germany) was used to detach the cells from the culture plate. Cells were maintained in a humidified incubator at 37 °C with 5% CO_2_.

### Preparation of quercetin

Quercetin hydrate [C15H10O7.xH2O] (Cat#337951, Sigma-Aldrich, St. Louis, MO, USA) was prepared in DMSO (Carl Roth GmbH + Co KG, Karlsruhe, Germany). DMSO at the corresponding final concentration was used as a control.

### Culture of A549 and H520 cells with quercetin

For the dose response to quercetin, a total of 4 × 10^5^ A549 and H520 cells each were plated in two separate 6-well plates and allowed to adhere to the wells for 24 h. After the initial 24 h period, the medium was replaced, and the cells were treated with different concentrations of quercetin hydrate (0,10, 50, 100 µM) for 48 h. At the end of the experiment, the cell count was determined using the Neubauer chamber slide, and the cells were subsequently resuspended in QIAzol (Cat#79306, QIAGEN, Erlangen, Germany). RNA was extracted using the Phenol-chloroform extraction method (Supplementary information). Further, RT-qPCR was performed to assess the expression of the genes involved in cell cycle arrest, CDK1, CDKN1A, RB1, and BAX, and oxidative stress genes: HO1 and NOQ1. A detailed list of the primer sequence is provided in Supplementary Table S1.

### Western blot

A549 cells were cultured under experimental conditions (see cell lines), yielding approximately 3 × 10^6^ cells per condition. After 24 h incubation, cells were treated with different doses of quercetin hydrate (0, 10 µM, 50 µM, and 100 µM) for the next 48 h. The cells were harvested and resuspended in 300 μl of M-PER RIPA lysis buffer (Cat#78501, Thermo-Fisher Scientific, Waltham, USA). List of antibodies, protein extraction, and the western blot process were carried out as described in Supplementary Table S2. Proteins were subsequently extracted, quantified using the Bradford assay (Cat#5000006, Bio-Rad, Hercules, CA, USA), and subjected to western blot analysis. Anti-GAPDH was used as a loading/housekeeping control; anti-p21 and anti-p53 were used as primary antibodies. The blot was developed and visualized with a Bio-Rad Chemic Doc system (Bio-Rad GmbH, Hercules, CA, USA). Results were analysed by Image Lab software using the lane and band tool. For each band, the software calculated the band volume and the background-corrected signal. With the results obtained, the target protein expression was normalised with the housekeeping control.

(i). $$N{ormalised\; expression}=\frac{{Target\; band\; intensisty}}{{Loading\; control\; intensity}}$$

### Immuno-fluorescence staining

A549 cells were seeded at a density of 4 × 10^5^ cells/well. After 24 h, the cells were treated with quercetin hydrate (10 µM, 50 µM, 100 µM) for the next 48 h. To concentrate cells onto a slide using the cyto-spin method, 5 × 10^4^ cells were suspended in 100 µl of PBS and then pipetted into the cyto-spin system. The samples were centrifuged in the cytocentrifuge (Thermo-Fisher Scientific-Waltman, MA, USA) for 5 min at 500 r.p.m. and were dried under a low-volume table hood (WALDNER Laboreinrichtungen GmbH & Co. KG, Wagen, Germany) for 24 h at RT. Further procedure is described in the Supplementary Information. DAPI stain was embedded in mounting medium: FluorSave^TM^ Reagent (Cat#4257082, Merck KG. A, Darmstadt, Germany), which stained the cell nuclei blue. EGFR and PD-L1 antibodies bound to the cell membrane were visualized in red with Alexa Fluor 555 and in green with Alexa Fluor 488, respectively. Details about the antibody dilution and staining procedure are described in Supplementary Table S3. For quantification, images were captured from 1 random field per condition at 20X magnification. Image analysis was performed using the ImageJ 1.52a software (National Institutes of Health, Maryland, USA). Corrected total cell fluorescence (CTCF) levels were quantified by measuring 5 individual cells per field. Regions of interest (ROIs) corresponding to individual cells were automatically selected using threshold-based segmentation, integrated density, and the area of selected cells were also measured for each ROI. To determine the background fluorescence, a background ROI was measured in a cell-free region. Corrected Total Cell Fluorescence (CTCF) was calculated using the formula:

(ii). *CTCF = Integrated Density – (Area of selected cell x Mean fluorescence of background)* [30]

### Flow cytometry

Under basal cell culture conditions, A549 cells express minimal baseline expression of PD-L1. To establish a detectable signal suitable for evaluating therapeutic intervention, A549 cells were stimulated externally with 50 ng/ml IFN-y for 24 h to upregulate the PD-L1 expression. After this cytokine-induced priming, the cells were treated with varying concentrations of quercetin hydrate (10 µM, 50 µM, 100 µM) for 24 h. At the end of the experiment, the A549 cells were harvested using Accutase (Cat#AC-EZ-0001, Anprotec, Bruckberg, Bavaria, Germany) and further processed for FACS in a 96-well U-bottom plate (Cat#163320, Thermo Scientific Nunclon^TM^ Delta Surface, Roskilde, Denmark). Flow cytometric analysis was performed to evaluate cell proliferation via Ki67 (1:50, Cat#151206, Biolegend, San Diego, CA, USA) and surface receptor expression of EGFR (1: 100, Cat#352908, Biolegend, San Diego, CA, USA) and PD-L1(1:50, Cat#557924, BD Pharmingen, Heidelberg, Germany). All centrifugation (Cat# 75004270, Thermo Fisher Scientific, Megafuge™ 16 Universal-Zentrifuge) steps were performed for 1 min at 2000 r.p.m., RT. Cells were first incubated with a viability marker: 575 V (Cat# 565694, BD Horizon, Heidelberg, Germany) for 15 min at RT to exclude dead cells. Prior to surface staining, cells were incubated with Fc block (Cat# 4222302, Biolegend, San Diego, CA, USA) for 10 min at RT to minimize non-specific antibody binding. For surface staining, cells were incubated with anti-human EGFR and anti-human PD-L1 antibodies (prepared in FACS buffer) for 20 min at 4°C. Following a washing step to eliminate unbound antibodies, the cells were fixed and permeabilized using a Fixation/ permeabilization kit (Cat#00522356, Invitrogen by Thermo-Fisher Scientific, Waltham, USA). Cells were then incubated with anti-human Ki67 antibody for 30 min in the dark at RT. For proper gating, single-stained controls as well as unstained cells were included in each experiment. Data acquisition was performed using a FACS Symphony A1 (BD) flow cytometer, and data were analysed using Flow cytometry software, Kaluza 2.3 (Beckman Coulter).

### Cell ROX staining

A549 and H520 cells were cultured on a 4-chamber slide (Cat#154526, Nunc™ Lab-Tek™ II Chamber Slide™ System, NY, USA) at a density of 1.5 × 10^5^ cells per chamber. On the following day, cells were treated with 50 µM quercetin hydrate for 48 h. For the assessment of oxidative stress, cells were incubated with the Cell ROX ^RM^ Green reagent (Lot# 3143106, Invitrogen by Thermo-Fisher Scientific, Waltham, USA) at a concentration of 5 µM for 30 min. Following the staining, cells were washed with PBS to get rid of additional stain, and the slide was mounted with a mounting medium (Lot#4161994, EMD Millipore Corp., USA), and fluorescence images were acquired using an AXIO Observer D1 Fluorescence microscope (Carl Zeiss, Munich, Germany) [31]. For quantification, images were captured from 4 random fields per condition at 20X magnification. Image analysis was performed using the ImageJ 1.52a software. CTCF was calculated using the formula described in Immuno-fluorescence staining.

### Wound healing assay

H520 cells and A549 cells were cultured in RPMI 1640 (supplemented with 10% FCS, 1% P/S, and 1% L-Glu) medium. A total of 2 × 10^5^ cells were seeded per well in a 24-well-plate and incubated for 24 h at 37 °C 5% CO_2_. After incubation, cells were treated with 5 µl of mitomycin C (Cat#M0503, Sigma-Aldrich, Darmstadt, Germany) for an hour. After incubation, 1 horizontal line was drawn across each well using a sterile 100 µl pipette tip, followed by 3x washing of each well with 1 ml sterile PBS (Anprotec, Cat# AC-BS-0002) [32]. After the washing step, RPMI with indicated concentrations of quercetin hydrate was added to the wells, and the plate was immediately transferred to the Keyence BZ-X800 microscope (Keyence GmbH, Frankfurt am Main, Germany) for the time-lapse to monitor wound closure. The BZ-X800 series software was utilized to convert the captured time-lapse images into videos for visualization (see Video in extended material). The scratch area was measured at 0 h and 20 h following treatment using ImageJ software. The resulting data was then imported into GraphPad Prism 9.0 for graph plotting.

### Transwell assay

Cell migration assay was performed utilizing 8 µm pore trans-well inserts (Cat# 665638, Greiner Bio-One ThinCert^TM^, Frickenhausen, Germany) in a 12-well cell culture plate (Cat# E23053D9, Greiner Cellstar®, Darmstatdt, Germany). 24 h prior to the assay, the A549 cells at approximately 70–80% confluency, were starved using a serum-free basal medium (RPM1 1640 supplemented with 1% P/S and 1% L-Glutamine) at 37 °C in a humidified chamber of 5% CO_2_ for 24 h.

Following incubation, the trans-well inserts were placed in the plate and pre-equilibrated with 300 µl of serum-free basal medium for 30 min in the incubator. The starved cells were processed for cell count and diluted to a final working density of 4 × 10^5^ cells/ml.

To evaluate the anti-migratory effects of quercetin, treatment groups were assigned as follows: negative control group (serum-free medium in both the upper and the lower chamber), positive control group, DMSO control group, and quercetin treatment groups (10 µM, 50 µM, 100 µM). 1.5 ml of complete growth medium (RPM1 1640 supplemented with 10% FCS, 1% P/S, and 1% L-Glutamine) was added to the lower chambers/wells of a 12-well plate to act as a chemoattractant. 200 µl of cell suspension with respective treatments was carefully transferred into the inserts that already contained 300 µl of serum-free medium (equivalent to 8 × 10^4^ cells/insert). The inserts were then carefully lowered into the wells containing complete medium. The plate was incubated at 37 °C in a humidified chamber of 5% CO_2_ for 24 h. Next, the media from the upper chambers was discarded, and the inserts were washed twice with PBS, and the cells were fixed with 1.5 ml of 4% PFA for 20 min at RT. The fixed cells were washed twice with PBS and permeabilized with 100% methanol for 30 min at RT. After 2 additional washes, the cell membranes were stained with 10% Giemsa staining solution (Cat#T8632.1, Carl Roth GmbH, Karlsruhe, Germany) prepared in Milli-pore water for 30 min at RT, followed by two final PBS washes. The inserts were allowed to dry for the next 30 min. To eliminate background signal during microscopy, the non-migrated cells inside the insert were thoroughly removed using damp and dry cotton swabs. The migrated cells on the lower surface of the membrane were visualized under an inverted microscope.

For quantification, images were captured from 5 random fields per insert at 40X magnification. The number of stained-migrated cells was quantified using the QuPath software (version 6). The mean migrated cells/group were calculated using the formula:

(iii). Mean migrated cells $=\frac{{Total}\,{cells}\,{counted}\,{across}\,{all}\,5\,{fields}\,}{5}$

### Murine models of lung cancer

C57BL/6JRj wild-type (WT) mice and BALB/cJRj WT mice were purchased from Janvier Laboratory. All experiments were undertaken with an approved license (no. Az 55.2-2532-2-1286-20) from the Ethical Committee of the Regierung Oberfranken (Erlangen, Germany). The mice were housed under pathogen-free conditions and underwent treatment at 9 weeks of age. Age-matched, same sex mice (female) were used in this study. Due to logistical constraints and the requirements for the same investigator to administer treatments and record physiological outcomes, investigators were not blinded to group allocation during animal experiments or the subsequent data analysis. Formal mathematical randomization was not used to allocate animals to experimental groups. Instead, animals were systematically assigned to ensure equal group sizes across all experimental conditions and to balance baseline characteristics.

### Quercetin treatment in a murine lung cancer model

All the mice were maintained as 5 mice per cage at a constant temperature with a 12 h dark or light cycle and on a standard mouse diet. In this murine model of lung cancer, the BALB/cJRj WT mice were injected into the tail vein with 2 × 10^5^ L1C2 cells on day 2.This established an experimental lung metastasis model, focusing primarily on the colonization phase of tumor progression. Quercetin was administered via oral gavage using a 20 G stainless steel feeding tube at a dose of 50 mg/kg every other day as noted in Fig. 6a, corresponding to an average daily dose of ~ 25 mg/kg/day. This dose was chosen based on the estimated daily quercetin intake in Western diets (25–30 mg/day) in humans [33]. Additional studies reported protective effects of quercetin when administered via oral gavage [34, 35]. The weight of the mice was monitored throughout the experiment Fig. 6a). The experiment was concluded, and the mice were sacrificed on day 20. A section of the lung was used for histology, and the remaining lung was processed to get the whole lung homogenate [32]. The inflammation score was evaluated across 3 H&E-stained lung cryosections. Tumor load determined during the study is listed in Supplementary Table S4. 300 mg of stool sample was collected from each mouse, and microbiome DNA isolation was conducted using the QIAmp Fast DNA stool kit (Cat#79306, Qiagen, Erlangen, Germany).

### Generation and culture of precision-cut lung slices (PCLS)

In the PCLS mouse model, an experimental lung metastasis model was established by injecting 5 × 10^5^ LL/2-luc-M38 cells into the tail vein of the C57BL/6JRj WT mice. The mice were sacrificed on day 10, and their lungs were excised to obtain PCLS. The PCLS method was previously described [36]. Mice were anaesthetized and euthanized according to the ethical guidelines. Following euthanasia, animals were secured to a dissecting board with their limbs extended. The fur was disinfected with 70% ethanol, and a portion of the fur was removed with anatomical scissors until the skin was exposed. A vertical incision was made above the thymus using a scalpel, and the tissue was carefully separated with tweezers to reveal the trachea and oesophagus. The trachea was then fixed on a flat surface of the spatula, and a small horizontal cut was made between two tracheal rings, and a cannula was inserted into the cut. A syringe filled with 1 ml of 2% low-melting agarose was inserted via the cannula and injected slowly while monitoring proper flow into the lung. The trachea was clamped at the incision site, and an ice-filled glove was placed on the lungs for a span of 10-15 min to allow the agarose to solidify. Medical yarn was threaded under the trachea and tied with a surgical knot to secure the tracheal tube. The clamp was removed, the rib cage was cut and lifted to access the lung lobes, which were then transferred to a 50 ml falcon filled with sterile cold PBS. The tissue slicer was set up with appropriate settings to process the lung tissue and obtain slices. The slices were collected in cold PBS buffer to maintain viability and transferred to cell culture plates.

The lung slices were washed 4x with PCLS medium for 30 min in a cell incubator maintained at 37 °C and 5% CO_2_. Following the washes, the PCLS were treated with 50 µM of quercetin hydrate and maintained in a humidified incubator for 48 h. Following 48 h, PCLS were processed for histology using Hematoxylin and Eosin staining (H&E) and RNA extraction. Supernatants from the cell culture samples were analyzed using LEGENDplex™ MU Inflammation Cytokine Panel (13-plex) w/VbP (Cat# 740446, San Diego, USA) in accordance with the manufacturer’s instructions.

### Hematoxylin and Eosin staining on murine paraffin-embedded lung

Lung lobes were excised, and a piece of it was fixed in 10% formalin-PBS solution, dehydrated, and embedded in paraffin. 5 µm-thick lung sections were cut from the paraffin blocks and stained with H&E to visualize lung tumors and inflammation. The stained sections were scanned using a digital scanner (Sysmex-P1000, 3DHistech, Germany, and Hamamatsu NanoZoomer S210, Hamamatsu Photonics, Japan). H&E staining was carried out in collaboration with the Institute of Pathology at the University Hospital of Erlangen. Lung sections are examined histologically by a pathologist from the Institute of Pathology at the University Hospital of Erlangen.

### Data analysis

Statistical analysis and data presentation for cell counts, qPCRs, western blots, ELISAs, and flow cytometric measurements were conducted using GraphPad Prism 9 software. The mean values and their standard errors were reported. Results were expressed with asterisks above lines connecting the corresponding values to indicate significance levels. The significance threshold was determined according to specified limits: * (one asterisk): *p* < 0.05 (indicates a statistically significant difference) ** (two asterisks): *p* < 0.01 (indicates a highly significant difference) *** (three asterisks): *p* < 0.001 (indicates a very highly significant difference) ns (not significant): *p* > 0.05 (indicates no statistically significant difference).

## Supplementary information

Supplementary Figure 1

Supplementary Figure 2

Supplementary Figure 3A - Uncropped Western blot image-GAPDH

Supplementary Figure 3B - Uncropped Western blot image-p21

Supplementary Figure 3C - Uncropped Western blot image- p53

Supplementary Figure 3D - Uncropped Western blot membrane image

Supplementary Figure 3E - Uncropped Western blot gel image

Supplementary Figure 4

Supplementary Figure 5

Supplementary Figure 6

Supplementary Figure 7

Supplementary Figure 8

Supplementary Figure 9

Supplementary Figure 10

Supplementary Figure 11

Supplementary Figure 12

Supplementary Figure 13

Original data

Supplementary information

Supplementary video S1a

Supplementary video S1b

Supplementary video S1c

Supplementary video S2a

Supplementary video S2b

Supplementary video S2c

Supplementary video S3a

Supplementary video S3b

Supplementary video S3c

Supplementary video S4a

Supplementary video S4b

Supplementary video S4c

## Data Availability

The microbiome raw 16S rRNA gene sequencing read files generated during this study have been deposited in the European Nucleotide Archive (ENA) at EMBL-EBI under the accession number PRJEB109998 (https://www.ebi.ac.uk/ena/browser/view/PRJEB109998). The transcriptomic raw mRNA sequencing read files of MACS-purified splenic CD8+Tcells generated during this study have been deposited in the European Nucleotide Archive (ENA) at EMBL-EBI under the accession number PRJEB110282. All other data are available in the article and its Supplementary files.

## Code availability

Codes for the imaging analysis are available in the Supplementary files.

## References

[1] Liu C, Yang D, Liu Y, Piao H, Zhang T, Li X, et al. The effect of ambient PM2.5 exposure on survival of lung cancer patients after lobectomy. Environ Health. 2023;22:23. 10.1186/s12940-023-00976-x. 36879322 PMC9990243

[2] Tahayneh K, Idkedek M, Abu Akar F. NSCLC: current evidence on its pathogenesis, integrated treatment, and future perspectives. J Clin Med. 2025;14:1025. 10.3390/jcm14031025. 39941694 PMC11818267

[3] Board, PATE Non-Small Cell Lung Cancer Treatment (PDQ®), <https://www.cancer.gov/types/lung/hp/non-small-cell-lung-treatment.

[4] Gioda A. Indoor air pollution from firewood combustion in Indigenous malocas in the Brazilian Amazon: exposure to fine particulate matter and associated health risks. J Bras Pneumol. 2025;51:e20250161. 10.36416/1806-3756/e20250161. 41172416 PMC12602047

[5] Rodak O, Peris-Díaz M, Olbromski M, Podhorska-Okołów M. Current landscape of non-small cell lung cancer: epidemiology, histological classification, targeted therapies, and immunotherapy. Cancers. 2021;13:4705. 10.3390/cancers13184705. 34572931 PMC8470525

[6] Araghi M, Mannani R, Heidarnejad Maleki A, Hamidi A, Rostami S, Safa SH et al. Recent advances in non-small cell lung cancer targeted therapy: an updated review. Cancer Cell Int. 2023;23. 10.1186/s12935-023-02990-y. PMC10416536 37568193

[7] Ullah A, Munir S, Badshah SL, Khan N, Ghani L, Poulson BG, et al. Important flavonoids and their role as therapeutic agents. Molecules. 2020;25:5243. 33187049 10.3390/molecules25225243 PMC7697716

[8] Batra P, Sharma AK. Anti-cancer potential of flavonoids: recent trends and future perspectives. 3 Biotech. 2013;3:439–59. 10.1007/s13205-013-0117-5. 28324424 PMC3824783

[9] Gricelis M, Michael RM, Juan BDS. Effects of flavonoids and its derivatives on immune cell responses. Recent Pat Inflamm Allergy Drug Discov. 2019;13:84–104. 10.2174/1872213X13666190426164124. 31814545

[10] Stavric B. Quercetin in our diet: from potent mutagen to probable anticarcinogen. Clin Biochem. 1994;27:245–8. 10.1016/0009-9120(94)90025-6. 8001284

[11] Reyes-Farias M, Carrasco-Pozo C. The anti-cancer effect of quercetin: molecular implications in cancer metabolism. Int J Mol Sci. 2019;20:3177. 31261749 10.3390/ijms20133177 PMC6651418

[12] Carullo G, Cappello AR, Frattaruolo L, Badolato M, Armentano B, Aiello F. Quercetin and derivatives: useful tools in inflammation and pain management. Future Med Chem. 2017;9:79–93. 10.4155/fmc-2016-0186. 27995808

[13] Carullo G, Perri M, Manetti F, Aiello F, Caroleo MC, Cione E. Quercetin-3-oleoyl derivatives as new GPR40 agonists: molecular docking studies and functional evaluation. Bioorg Med Chem Lett. 2019;29:1761–4. 10.1016/j.bmcl.2019.05.018. 31104992

[14] Carrasco-Pozo C, Tan KN, Reyes-Farias M, De La Jara N, Ngo ST, García-Díaz DF, et al. The deleterious effect of cholesterol and protection by quercetin on mitochondrial bioenergetics of pancreatic beta-cells, glycemic control and inflammation: In vitro and in vivo studies. Redox Biol. 2016;9:229–43. 10.1016/j.redox.2016.08.007. 27591402 PMC5011185

[15] Guo Y, Bruno RS. Endogenous and exogenous mediators of quercetin bioavailability. J Nutrit Biochem. 2015;26:201–10. 10.1016/j.jnutbio.2014.10.008. 25468612

[16] Ozaki T, Nakagawara A. Role of p53 in cell death and human cancers. Cancers. 2011;3:994–1013. 24212651 10.3390/cancers3010994 PMC3756401

[17] Tanigawa S, Fujii M, Hou DX. Stabilization of p53 is involved in quercetin-induced cell cycle arrest and apoptosis in HepG2 cells. Biosci Biotechnol Biochem. 2008;72:797–804. 10.1271/bbb.70680. 18323654

[18] Karlsen E-A, Kahler S, Tefay J, Joseph SR, Simpson F. Epidermal growth factor receptor expression and resistance patterns to targeted therapy in non-small cell lung cancer: a review. Cells. 2021;10:1206. 34069119 10.3390/cells10051206 PMC8156654

[19] Cui J-W, Li Y, Yang Y, Yang H-K, Dong J-M, Xiao Z-H, et al. Tumor immunotherapy resistance: revealing the mechanism of PD-1/PD-L1-mediated tumor immune escape. Biomed Pharmacother. 2024;171:116203. 10.1016/j.biopha.2024.116203. 38280330

[20] Lan Y, Dong C, Wu M, Yuan R, Yang K, Yang Z, et al. Quercetin ameliorates epithelial-mesenchymal transition and inflammation by targeting FSTL1 and modulating the NF-kappaB pathway in pulmonary fibrosis. Front Pharmacol. 2025;16:1594757. 10.3389/fphar.2025.1594757. 40808692 PMC12343526

[21] Mukai R, Fujikura Y, Murota K, Uehara M, Minekawa S, Matsui N et al. Prenylation enhances quercetin uptake and reduces efflux in Caco-2 cells and enhances tissue accumulation in mice fed long-term. J Nutr. 2013;143. 10.3945/jn.113.176818. 23902958

[22] Conquer J, Maiani G, Azzini E, Raguzzini A, Holub B. Supplementation with quercetin markedly increases plasma quercetin concentration without effect on selected risk factors for heart disease in healthy subjects. J Nutr. 1998;128:593–7. 10.1093/jn/128.3.593. 9482769

[23] Lan H, Hong W, Qian D, Peng F, Li H, Liang C, et al. Quercetin modulates the gut microbiota as well as the metabolome in a rat model of osteoarthritis. Bioengineered. 2021;12:6240–50. 10.1080/21655979.2021.1969194. 34486477 PMC8806632

[24] Bai Q, Zhao Z, Duan Y, Cai R, Chen Y, Zhou C, et al. Enrichment of short-chain fatty acid-producing bacteria by pH-responsive sodium alginate and chitosan-encapsulated quercetin. Front Microbiol. 2025;16:2025. 10.3389/fmicb.2025.1594012. PMC12364867 40842836

[25] Bhunia A, Anbazhagan GK, Babu D, STG & Lokesh E. Short-chain fatty acids (SCFAs) in gut health: Implications for drug metabolism and therapeutics. Med Microecology. 2025;25. 10.1016/j.medmic.2025.100139.

[26] Thome CD, Tausche P, Hohenberger K, Yang Z, Krammer S, Trufa DI, et al. Short-chain fatty acids induced lung tumor cell death and increased peripheral blood CD4+ T cells in NSCLC and control patients ex vivo. Front Immunol. 2024;15:1328263. 10.3389/fimmu.2024.1328263. 38650948 PMC11033355

[27] Shen S, Wu Y, Shao Z, Li Y, Peng D, Li B, et al. LTF as a potential predictive biomarker for durable benefit from first-line chemo-immunotherapy in small cell lung cancer. Cancer Sci. 2025;116:1522–36. 10.1111/cas.70049. 40095278 PMC12127099

[28] A Jewett BB. Interferon-alpha activates cytotoxic function but inhibits interleukin-2-mediated proliferation and tumor necrosis factor-alpha secretion by immature human natural killer cells. J Clin Immunol. 1995. 10.1007/BF01489488. 7759599

[29] Heim L, Chiriac M, Kachler K, Mitsch S, Yang Z, Kölle J et al. IL-9 Producing tumor-infiltrating lymphocytes (Til) and Treg subsets drive immune escape of tumor cells in non-small cell lung cancer. SSRN Electron J. 2021. 10.2139/ssrn.3944543. PMC9065342 35514957

[30] El-Sharkawey A. Calculate the corrected total cell fluorescence (CTCF). Quantitative measurement of cellular fluorescence from microscopy images using ImageJ and corrected total cell fluorescence (CTCF). 2016. 10.13140/RG.2.1.1307.8008.

[31] Steppuhn H, Hohenberger K, Mittler S, Trump S, Carvalho C, Rauh M et al. 4-Octyl Itaconate ameliorates diesel exhaust particle-induced oxidative stress in nasal epithelial cells. Front Immunol. 2025;16. 10.3389/fimmu.2025.1640499. PMC12411186 40918100

[32] Harre P, Hohenberger K, Krammer S, Yang Z, Tausche P, Willar J, et al. Dietary ω−3 polyunsaturated fatty acids (PUFAs) reduce cholesterol-driven non-small cell lung cancer (NSCLC) progression in mouse models of disease. Commun Med. 2025;5:432. 10.1038/s43856-025-01193-y. 41131338 PMC12549838

[33] Russo GL. Ins and outs of dietary phytochemicals in cancer chemoprevention. Biochem Pharmacol. 2007;74:533–44. 10.1016/j.bcp.2007.02.014. 17382300

[34] Duarte J, Jiménez R, O’Valle F, Galisteo M, Pérez-Palencia R, Vargas F et al. Protective effects of the flavonoid quercetin in chronic nitric oxide-deficient rats. J Hypertens. 2002;20:1843–54. 10.1097/00004872-200209000-00031 12195128

[35] Duarte J, Pérez-Palencia R, Félix V, Ocete M, Pérez-Vizcaíno F, Zarzuelo A, et al. Antihypertensive effect of the flavonoid quercetin in spontaneously hypertensive rats. Br J Pharmacol. 2001;133:117–24. 11325801 10.1038/sj.bjp.0704064 PMC1572775

[36] Tatcheff M, Carvalho C, Willar J, Nendel E, Krammer S, Chiriac MT, et al. Analyzing pathophysiology and immune cells and their cytokines and mediators in precision-cut slices of the murine lung. Curr Protoc. 2025;5:e70087. 10.1002/cpz1.70087. 39873159 PMC11773432

