# Supplementary material for: Immunoregulatory role of quercetin in lung cancer
Source: Cell Death Discov. 2026 Sep 14;12:372. doi: 10.1038/s41420-026-03312-7 (PMC13574831; doi:10.1038/s41420-026-03312-7)
Supplement: Supplementary file 1 [file 41420_2026_3312_MOESM19_ESM.docx]

**Supplementary information**

**Immunoregulatory role of Quercetin in lung cancer**

Christine Carvahlo ^1^, Elvedina Nendel ^1^, Susanne Mittler ^1^, Sonja Trump ^1^, Adriana Geiger ^1^, Jonas Willar ^1^, Mircea T. Chiriac ^2^, Stefan Wirtz ^2^, Carol I. Geppert ^3,4,5^ and Susetta Finotto *^1,4,5,6^

^1^ Department of Molecular Pneumology, Friedrich Alexander University Erlangen-Nürnberg (FAU), Universitätsklinikum Erlangen, Erlangen, Germany.

^2^ Department of Internal Medicine 1, Friedrich Alexander University Erlangen-Nürnberg (FAU), Universitätsklinikum Erlangen, Erlangen, Germany.

^3^ Institute of Pathology, University Hospital, Friedrich-Alexander-Universität Erlangen-Nürnberg, Erlangen, Germany

^4^Bavarian Cancer Research Center (BZKF), Erlangen, Germany

^5^ Comprehensive Cancer Center Erlangen-EMN (CCC ER-EMN), Erlangen, Germany

^6^ Deutsches Zentrum für Immuntherapie (DZI), Erlangen, Germany

***Corresponding Author:**

Susetta Finotto, PhD,

Laboratories of Cellular and Molecular Lung Immunology,

Department of Molecular Pneumology, Friedrich-Alexander-Universität (FAU) Erlangen-Nürnberg, Erlangen, Germany

Hartmannstraße 14,

91052 Erlangen, Germany.

Phone: +49 9131-85-35883;

E-mail:  [Susetta.Finotto@uk-erlangen.de](mailto:Susetta.Finotto@uk-erlangen.de)

**Supplementary information**

**RNA isolation from PCLS and cell lines, Reverse transcription (RT) and Quantitative Real-Time PCR (qPCR)**

Total RNA isolated from the PCLS was stored at -80 ^o^C in 500 µl of QIAzol® Lysis Reagent (Cat# 79306, Qiagen, Erlangen, Germany) using homogenization and the Phenol-chloroform method. Briefly, the PCLS were homogenized in a homogenizer (Analytik Jena, Jena, Germany) using 2 metallic beads (Cat#845-CS-11400050, Innuscreen GmbH, Berlin, Germany) of size 1.4-1.6 mm for 1 minute. Further, the beads were removed and RNA isolation was carried out using the Phenol-chloroform method. RNA concentration and purity was determined using a NanoDrop™ spectrophotometer (Thermo Fisher Scientific, Waltman, USA). cDNA was synthesized according to the manufacturer’s protocol using the Revert-Aid First Strand cDNA Synthesis Kit (Thermo-Fisher Scientific, Lithuania) that included: Buffer (Lot#3281692), Ribo-Lock RNase Inhibitor (Cat#EO0381), dNTP Mix (Cat#R0192), Reverse Transcriptase (Cat#EP0441) and Random Hexamer Primer (Cat#SO142). qPCR was performed with iTaq Universal SYBR Green Supermix (Bio-Rad Laboratories, Cat# #1725124, München, Germany) using the CFX-96 Real-Time PCR Detection System (Bio-Rad Laboratories, Hercules, CA, USA). The primers used were ordered from Eurofins Genomics (Ebersberg, Germany) and the respective sequences are shown in **Supplementary** **Table S1**. The gene expression of all analyzed genes from the murine lung sections were normalized on the housekeeping gene mHPRT and that of the A549 and H520 cell lines were normalized on the housekeeping gene hRLP30, using the ΔCt method. All reactions were performed in duplicates.

**Supplementary Table S1: qPCR-Primer**

Fwd = forward Rev = reverse m = murine h = human

| **PRIMER** | **Sequence (5´– 3´)** |
| --- | --- |
| hBAX | Fwd: GCCCTTTTGCTTCAGGGTTTCA  Rev: GCAGGGTAGATGAATCGGGG |
| hCDK1 | Fwd: ACTACAGGTCAAGTGGTAGC  Rev: TCCCGAATTGCAGTACTAGG |
| hCDKN1A | Fwd: CTGGGGATGTCCGTCAGAAC  Rev: CATTAGCGCATCACAGTCGC |
| hHmox1 | Fwd: CCAGGCAGAGAATGCTGAGTTC  Rev: AAGACTGGGCTCTCCTTGTTGC |
| hNqo1 | Fwd: CCTGCCATTCTGAAAGGCTGGT  Rev: GTGGTGATGGAAAGCACTGCCT |
| hRb1 | Fwd: CAGAAGGTCTGCCAACACCAAC  Rev: TTGAGCACACGGTCGCTGTTAC |
| hRLP30 | Fwd: CTGGTGTCCATCACTACAGTGG  Rev: CCAGTCTGTTCTGGCATGCTTC |
| mBAX | Fwd: GCCTTTTTGCTACAGGGTCAT  Rev: TTCATCTCCAATTCGCCGGA |
| mCDK1 | Fwd: CATGGACCTCAAGAAGTACCTGG  Rev: CAAGTCTCTGTGAAGAACTCGCC |
| mCDKN1A | Fwd: TCGCTGTCTTGCACTCTGGTGT  Rev: CCAATCTGCGCTTGGAGTGATAG |
| mHmox1 | Fwd: CACTCTGGAGATGACACCTGAG  Rev: GTGTTCCTCTGTCAGCATCACC |
| mHPRT | Fwd: GCC CCA AAA TGG TTA AGG TT  Rev: TTG CGC TCA TCT TAG GCT TT |
| mNQO1 | Fwd: GCCGAACACAAGAAGCTGGAAG  Rev: GGCAAATCCTGCTACGAGCACT |
| mRb1 | Fwd: CCTTGAACCTGCTTGTCCTCTC  Rev: CTGAGGCTGCTTGTGTCTCTGT |

**Western Blot:**

A549 cultured cells were harvested and lysed directly in ice-cold M-PER RIPA lysis buffer (Cat#78501, Thermo-Fisher Scientific, Waltham, USA) containing protease inhibitors. Cell suspensions were incubated on ice for 30 minutes, and centrifuged at 14,000 x g for 15 minutes at 4°C to remove debris. The supernatant containing soluble proteins was collected for analysis. Protein concentration from cell lysates were quantified using the Bradford assay (Cat#5000006, Bio-Rad, USA). Samples were denatured at 95°C for 5 minutes in 4X LDS Sample Buffer (Cat#NP0007, Thermo-Fisher Scientific, Waltham, USA) and 1 M dithiothreitol (DTT) (Cat#P2325, Thermo-Fisher Scientific, Waltham, USA). Equal amounts of protein (20 µg per lane) were loaded on Mini-PROTEAN TGX Stain-Free Gel (Cat#4568086, Bio-Rad, Hercules, CA, USA) using the running buffer at 120 V for 1 hour and transferred on a 0.2 µm nitrocellulose membrane-Trans-Blot Turbo Transfer Pack (Cat#1704158, Bio-Rad, Hercules, CA, USA) using the Trans- Blot Turbo Transfer System (Bio Rad, GmbH, Hercules, CA, USA). The gel and the blot were visualized using the Chemi-Doc Imaging System (Bio-Rad GmbH, Hercules, CA, USA). Following protein transfer, blots were blocked for 1 hour at RT with 3% TBST/Milk (Cat#T145.3, Carl Roth GmbH, Karlsruhe, Germany) followed by 3x washing step with TBST buffer (Cat#4855.2, Tris-buffer + Cat#9127.1, Tween20; Carl Roth GmbH, Karlsruhe, Germany). The blot was further incubated overnight at 4°C with primary antibodies against p21 and p53 (diluted in blocking buffer); and GAPDH (diluted in 5% BSA). Membranes were washed and further incubated with appropriate HRP-conjugated secondary antibodies (anti-mouse or anti-rabbit) for 1 hour at RT. Following the washing step, the blot was developed using SuperSignal™ West Femto substrate (Cat# 34095, Thermo-Scientific, Waltham, USA) and visualised with the Chemi-Doc Imaging System. Band intensities were quantified using ImageLab software (Bio-Rad) and normalized to total protein levels. The membranes were further stripped with Restore^TM^ Western Blot Stripping solution (Cat#UK290782, Thermo-Fisher Scientific, Waltham, USA) for 15 minutes at RT and subsequently incubated with the next primary antibody.

**Supplementary Table S2**

| **Antibody** | **Dilution** | **Company** | **Catalog No.** |
| --- | --- | --- | --- |
| Anti-mouse | 1:100 | Cell signaling Technology | 7076S |
| Anti-GAPDH | 1:1000 | Cell signaling Technology | 2118S |
| Anti-p21 | 1:200 | Santa Cruz Biotechnology | H3023 |
| Anti-p53 | 1:500 | Santa Cruz Biotechnology | E1124 |
| Anti-Rabbit | 1:100 | Cell signaling Technology | 7074S |

**Immunohistochemistry (IHC)**

Cyto-spin preparations were fixed in 4% PFA (Cat# 0335.1, Roth) prepared in PBS for 10 minutes at RT followed by 3x washing with PBS. Cells were permeabilized using Perm buffer composed of 0.25% Triton X (Cat# 3051.3, Roth, Karlsruhe, Germany) and PBS (Cat# AC-BS-0002, Anprotec), followed by the washing step. Non-specific binding was blocked using blocking buffer composed of 5% BSA (Cat# 11930.03, Serva, Heidelberg, Germany) in PBS for 30 minutes. Primary antibodies were prepared in 1% BSA in PBS and the secondary antibodies were prepared in PBS.

**Supplementary Table S3:**

| **Time** | **Antibody** | **Host species** | **Dilution** | **Company** |
| --- | --- | --- | --- | --- |
| 1h | EGFR | Rabbit polyclonal | 1:100 | eBioscience (Cat#14-6747-81) |
| 3x PBS |  |  |  |  |
| 1h | Alexa Fluor 555 | Rabbit polyclonal | 1:500 | Invitrogen (Cat# A-21430) |
| 3x PBS |  |  |  |  |
| 1h | PD-L1 | Mouse monoclonal | 1:50 | Cell signalling (Cat#29122) |
| 3x PBS |  |  |  |  |
| 2h | Alexa Fluor 488 | Mouse monoclonal | 1:500 | Invitrogen (Cat# A-10684) |
| 3x PBS |  |  |  |  |

Slides were mounted using the FluorSave mounting medium (REF 345789, Merck, Darmstadt, Hesse, Germany) containing DAPI for nuclear counterstaining. Fluorescence images were acquired using a Axio Observer D1 fluorescence microscope (40X) (Zeiss, Oberkochen, Baden-Württemberg, Germany) under identical exposure settings for all samples.

**Supplementary Table S4:**

| **Treatment** | **Mice No.** | **Tumor load% of area of lung** |
| --- | --- | --- |
| Oil | T4 | 0 |
|  | T5 | 10 |
|  | T6 | 0 |
| Quercetin | T1 | 0 |
|  | T2 | 0 |
|  | T3 | 8 |

**Quercetin supplementation modulates the systemic immune response to lung cancer**

In this murine model for lung cancer, the BALB/cJRj WT mice were injected in the tail vein with 5x10^5^ L1C2 cells on day 0. Quercetin was solubilized in olive oil and was administered via oral gavage using a 20G stainless steel feeding tube at a dose of 50 mg/kg every other day as noted in **Fig. 9a.** The mice in the control group received olive oil via oral gavage. All the mice were maintained on standard mouse diet. This experimental setup was designed to evaluate the therapeutic potential of quercetin in comparison to the control group.

**Isolation of total splenic cells and CD8+ cells sorting**

Spleen single-cell suspensions were generated by mincing spleen tissue into small fragments, which was passed through a 40 µm cell strainer (Cat#542040, Greiner Bio-One). Cells were centrifuged (10 minutes, 1500 r.p.m., 4 °C), supernatant was discarded, and pellet was resuspended in ACK-lysis buffer (Preparation: 0.15 M NH4Cl - Cat# P726.2, Carl Roth GmbH + Co. KG; 0.01 M KHCO3 - Cat#P748.1, Carl Roth GmbH + Co. KG; 100 M Na2EDTA - Cat# 1034.1000, GERBU Biotechnik GmbH; dissolved and sterile filtered in deionized H_2_O; pH=7.2–7.4). After 2 minutes of incubation, cells were centrifuged again under the same conditions. The supernatant was discarded, and cells were resuspended in PBS. To eliminate fat, the suspension was transferred into a 10 ml pipette and gently dispensed into a Falcon tube, allowing the fat to stick to the pipette walls. This step was followed by centrifugation (5 minutes, 1500 r.p.m., 4 °C). The pellet was resuspended in 10 ml PBS, and viable cell numbers were determined using Trypan blue staining in a Neubauer counting chamber.

CD8+ T cells were isolated by positive selection using MACS CD8a (Ly-2) Micro-Beads (Cat# 130-117-044, Miltenyi Biotec, Bergisch Gladbach, Germany) following the manufactures instructions.

**Supplementary Table S5:**

| **Gene** | **Description** |
| --- | --- |
| Pou6f1 | Driver of tumor proliferation and a viable therapeutic target in clear cell adenocarcinoma ^1^ |
| Celsr1 | Oncogene promoting glioma cell proliferation, migration, and invasion^2^ |
| St3gal6 | Increased St3gal6 was positively correlated to tumor stage^3^ |
| Lcn2 | High levels of Lcn2 have been associated with increased cell proliferation, angiogenesis, cell invasion, and metastasis^4^ |
| Ltf | Tumor suppressor in small cell lung cancer. Ltf expression is associated with improved chemo-immunotherapy outcomes^5^ |
| Sik2 | SIKs actively participate in regulating the invasive function of breast cancer cells and influence the clinical course of breast cancer patients^6^ |
| Adnp | Adnp is associated with immune infiltration and radiosensitivity in hepatocellular carcinoma^7^ |
| Jun | Increased Jun expression in response to certain agonists (e.g., PAUF, CSF1) in TME cells can result in unfavourable effects including increased cancer cell migration or release of immunosuppressive cytokines^8^ |
| Trnp | Trnp gene encodes a novel mammalian conserved nuclear protein that can accelerate cellcycle progression^9^ |
| Ms4a6c | Primarily identified as a marker of tumor-associated myeloid cells^10^ |
| Gm75004 | No data available |
| Gm67332 | No data available |

**RNA isolation and RNA sequencing**

To study the transcriptional landscape, total RNA was extracted from MACS sorted spleen CD8+ T cells, preserved in RNA stabilization solution (RLT buffer) and stored at -80°C. RNA extraction was carried out using the RNeasy Mini kit (Cat#74106, Qiagen, Hilden, Germany). Quality control and sequencing were conducted by Novogene Corporation Inc. RNA integrity was checked using an Agilent Bioanalyzer, excluding samples with an RNA Integrity Number (RIN) below 7. The libraries were prepared using a PolyA enrichment method for bulk mRNA sequencing which involved enriching mRNA using poly-T oligo-attached magnetic beads, followed by mRNA fragmentation and cDNA synthesis using random hexamer primers and dTTP for a non-directional library. Library quality was assessed using Qubit, real-time PCR, and Bioanalyzer. Sequencing was performed on NovaSeq X Plus platform using a PE150 sequencing strategy. Raw sequencing data underwent quality control using FastQC, including adapter and low-quality base trimming with Trimmomatic. Reads were aligned to the mouse reference genome (*Mus Musculus*, GRCm39/mm39).

**Supplementary References**

1	Suzuki, N., Yoshioka, N., Uekawa, A., Matsumura, N., Tozawa, A., Koike, J. *et al.* Transcription Factor POU6F1 Is Important for Proliferation of Clear Cell Adenocarcinoma of the Ovary and Is a Potential New Molecular Target. *International Journal of Gynecological Cancer* **20**, 212–219 (2010). <https://doi.org:https://doi.org/10.1111/IGC.0b013e3181c97ae0>

2	Wang, G., Li, Y., Zhang, D., Zhao, S., Zhang, Q., Luo, C. *et al.* CELSR1 Acts as an Oncogene Regulated by miR-199a-5p in Glioma. *Cancer Manag Res* **12**, 8857–8865 (2020). <https://doi.org:10.2147/CMAR.S258835>

3	Dalangood, S., Zhu, Z., Ma, Z., Li, J., Zeng, Q., Yan, Y. *et al.* Identification of glycogene-type and validation of ST3GAL6 as a biomarker predicts clinical outcome and cancer cell invasion in urinary bladder cancer. *Theranostics* **10**, 10078–10091 (2020). <https://doi.org:10.7150/thno.48711>

4	Santiago-Sanchez, G. S., Pita-Grisanti, V., Quinones-Diaz, B., Gumpper, K., Cruz-Monserrate, Z. & Vivas-Mejia, P. E. Biological Functions and Therapeutic Potential of Lipocalin 2 in Cancer. *Int J Mol Sci* **21** (2020). <https://doi.org:10.3390/ijms21124365>

5	Shen, S., Wu, Y., Shao, Z., Li, Y., Peng, D., Li, B. *et al.* LTF as a Potential Predictive Biomarker for Durable Benefit From First-Line Chemo-Immunotherapy in Small Cell Lung Cancer. *Cancer Science* **116**, 1522–1536 (2025). <https://doi.org:https://doi.org/10.1111/cas.70049>

6	Wu, Q., Qu, C., Xin, L., Yang, F. & Xu, L. RETRACTED: SIK2-positive tumor epithelial cells in breast cancer maybe potential anti-cancer messengers: A systematic analysis from a single-cell perspective. *Environmental Toxicology* **39**, 768–782 (2024). <https://doi.org:https://doi.org/10.1002/tox.23987>

7	Wang, X., Peng, H., Zhang, G., Li, Z., Du, Z., Peng, B. *et al.* ADNP is associated with immune infiltration and radiosensitivity in hepatocellular carcinoma for predicting the prognosis. *BMC Med Genomics* **16**, 178 (2023). <https://doi.org:10.1186/s12920-023-01592-x>

8	Jafri, Z., Li, Y., Zhang, J., O'Meara, C. H. & Khachigian, L. M. Jun, an Oncological Foe or Friend? *Int J Mol Sci* **26** (2025). <https://doi.org:10.3390/ijms26020555>

9	Volpe, M., Shpungin, S., Barbi, C., Abrham, G., Malovani, H., wides, R. *et al.* trnp: A Conserved Mammalian Gene Encoding a Nuclear Protein That Accelerates Cell-Cycle Progression. *DNA and Cell Biology* **25**, 331–339 (2006). <https://doi.org:10.1089/dna.2006.25.331>

10	Silva-Gomes, R., Mapelli, S. N., Boutet, M. A., Mattiola, I., Sironi, M., Grizzi, F. *et al.* Differential expression and regulation of MS4A family members in myeloid cells in physiological and pathological conditions. *J Leukoc Biol* **111**, 817–836 (2022). <https://doi.org:10.1002/JLB.2A0421-200R>

**Supplementary figure legends**

**Supplementary Figure S1: Expression of cell cycle and apoptotic genes in A549 cells**

(a) Relative mRNA expression of CDK1 gene measured by qPCR and normalized to housekeeping control (RLP30) in the A549 cell line. Data is presented as mean with SD from n=3 independent experiments. Statistical significance was assessed by Brown-Forsythe ANOVA test, p< 0.05 was considered significant.

**Supplementary Figure S2: Expression of cell cycle and apoptotic genes in H520 cells**

(a) Quantification of live and dead H520 cells determined using trypan blue exclusion. (b) Relative mRNA expression of genes involved in cell cycle: CDK1, CDKN1A and BAX measured by qPCR and normalized to housekeeping control (RLP30) in the H520 cell line. Data is presented as mean with SD from n=3 independent experiments. Statistical significance was assessed by Brown-Forsythe ANOVA test, p< 0.05 was considered significant.

**Supplementary Figure S3: Representative western blot images**

Uncropped original western blot images corresponding to the cropped blots shown in Figure 1B(c). (A)Membrane was probed with antibody against GAPDH which was used as a loading control/housekeeping gene. (B)Membrane was probed with antibody against p21 which was used as a primary antibody. (C)Membrane was probed with antibody against p53 which was used as a primary antibody. (D) Western blot image acquired using Bio-Rad Chemi-Doc imaging system. (E) Western blot Gel image acquired using Bio-Rad Chemi-Doc imaging system. Lane order is identical to that presented in the main figure. No additional image adjustments were applied other than uniform contrast and brightness.

**Supplementary Figure S4: Expression of cell cycle and oxidative stress genes in A549 cells**

(a) Quantification of dead A549 cells determined using trypan blue exclusion. (b) Relative mRNA expression of genes involved in cell cycle: CDK1 and BAX were measured by qPCR and normalized to housekeeping control (RLP30). (c) Relative mRNA expression of genes related to oxidative stress: HO1 and NQO1 were measured by qPCR and normalized to housekeeping control (RLP30). Data is presented as mean with SD from n=3 independent experiments. Statistical significance was assessed by unpaired t-test, p< 0.05 was considered significant.

**Supplementary Figure S5: Expression of cell cycle and oxidative stress genes in H520 cells**

(a) Quantification of the dead H520 cells determined using trypan blue exclusion. (b) Relative mRNA expression of CDK1, RB1 and BAX were measured by qPCR and normalized to housekeeping control (RLP30). (c) Relative mRNA expression of genes related to oxidative stress: NQO1were measured by qPCR and normalized to housekeeping control (RLP30). Data is presented as mean with SD from n=3 independent experiments. Statistical significance was assessed by unpaired t-test, p< 0.05 was considered significant.

**Supplementary Figure S6: Flow cytometry gating strategy**

Sequential gating strategy used to analyse surface receptor expression and cell proliferation following treatment. Debris were excluded and total A549 cell population was identified using forward side scatter (FSC) and side scatter (SSC). Single cells (singlets) were isolated by using FSC-Area (A) against FSC-Height (H) to eliminate doublets. Cell viability was determined using live/dead staining. Representative dot plots demonstrate the gating boundaries used to determine the positive (+) and negative (-) populations of EGFR, PD-L1 and Ki67 expression gated on the live A549 cell population.

**Supplementary Figure S7: ROS generation in H520 cells detected by Cell-ROX**

H520 cells were treated with 50 µM of quercetin for 48 hours and later stained with Cell ROX reagent to detect ROS. (a) Representative fluorescence microscopy images captured at 20X magnification from 4 random fields per condition showing ROS in green. (b) Quantification of CTCF measured in 5 individual cells per field. Data are presented as mean with SD. Statistical significance was assessed by unpaired t-test, p< 0.05 was considered significant.

**Supplementary Figure S8: Quantification of tumor cell migration using Image J**

(a) Wound closure measured at 0 h following treatment using Image J software. (b) Wound closure measured at 20 h following treatment using Image J software. Wound area was manually outlined using the polygon selection tool in the cell free area. Gap area was quantified using: Analyse🡪Measure. The raw gap area (µm^2^) values were used for statistical analysis and graphical representation without normalization to percentage wound closure. (c) Representative image and quantitative analysis of cell migration following treatment: Number of migrated A549 cells were manually quantified using Image J software. To ensure accuracy, oval selection tool was utilized to individually mark and count each migrated cell across five random fields per insert or condition.

**Supplementary Figure S9: Assessment of tumor burden and inflammation score**

(a) Tumor burden was evaluated by measuring tumor load (by Carol I. Geppert). (b) Inflammation score was performed by visual assessment of the tissue sections (by Carol I. Geppert). The inflammation score ranged from 1-5, which represented the degree of alveolar infiltration and bronchial inflammation.

**Supplementary Figure S10: Quercetin increased abundance SCFA producing bacteria in the gut**

(a) Bar plot depicting the total number of biological samples analysed withing each experimental group. (b) Distribution of sequence reads (library size) obtained per sample across groups. (c) PCoA plot based on the Bray-Curtis dissimilarity metric. (d) Profiling of microbial richness and evenness within individual samples across experimental cohorts; standard metrics such as observed richness, fisher diversity, Shannon diversity and Inverse Simpson diversity are presented to display overall species distribution. (e) Relative abundance of the bacterial taxa in stool samples of mice at the Genus, Family, Order, Class and Phylum level determined by the 16s rRNA sequencing.

**Supplementary Figure S11: Transcriptomic heatmap of splenic CD8+ Tcells**

(a) BALB/c mice were injected with 5x10^5^ L1C2 tumor cells on day 0 to induce lung tumor Quercetin (50 mg/kg/day) was administered via oral gavage according to the experimental scheme *[Created in BioRender].* Mice in the control group (n=4) and the quercetin treated group (n=4) were maintained on the standard diet. Mice in the control group received no additional treatment. (b) The heat-map displays a subset of 12 representative genes significantly modulated by the quercetin treatment. Columns represent individual biological replicates (Control group -Standard diet: T3-T5 (n=3), Quercetin: T21, T23, T24 (n=3)); and rows represent individual genes. To account for data consistency, one outlier biological replicate was excluded from each group (leaving n=3 sample size in both the groups). Red indicates up-regulation and purple represents down-regulation.

**Supplementary Figure S12: Gene expression analysis in PCLS from tumor-induced and naive WT mice**

PCLS from both tumor and naive mice (WT) were processed for RNA extraction. (a) Relative mRNA expression of genes involved in cell cycle: CDK1, RB1 and BAX were measured by qPCR and normalized to housekeeping control (HPRT) in the tumor-PCLS. (b) Relative mRNA expression of genes related to oxidative stress: HO1 and NQO1measured by qPCR and normalized to housekeeping control (HPRT) in the tumor-PCLS. (c) Relative mRNA expression of genes involved in cell cycle: CDK1, RB1 and BAX were measured by qPCR and normalized to housekeeping control (HPRT) in the naive-PCLS. (d) Relative mRNA expression of genes related to oxidative stress: HO1 and NQO1measured by qPCR and normalized to housekeeping control (HPRT) in the naive- PCLS. Data is presented as mean with SD from n=3 independent experiments. Statistical significance was assessed by unpaired t-test, p< 0.05 was considered significant.

**Supplementary Figure S13: Cytokine analysis in PCLS supernatant**

48 hours after quercetin (50 µM) treatment, supernatants were collected and analysed using LEGENDplex™ MU Inflammation Cytokine Panel (13-plex, Cat# 740446) kit and samples were acquired in BD FACS Symphony^TM^ A1 and analysed in Kaluza Analysis 2.1 (Beckman Coulter). (a) Measured beads were divided into population A and B depending on their size and granularity and were then subdivided into their corresponding populations representing the specific cytokines. Mean fluorescence intensity (MFI) in the APC and PE channel was used to quantify the cytokine concentration. (b) Murine serum IL-1b level [pg/ml]. (c) Murine serum IFN-y level [pg/ml]. (d) Murine serum IL-6 level [pg/ml]. (e) Murine serum TNF-a level [pg/ml]. (f) Murine serum MCP-1 level [pg/ml].

**Supplementary Videos**

**Supplementary Video S1: Representative wound healing assay of A549 control cells.**

Time-lapse phase contrast microscopy showing the migration of A549 cells in the control group over a period of 20 hours. Images in the selected region were captured every 20 minutes using Keyence BZ-X800 series microscope. The time lapse images were converted into videos (960 x 720 pixel) using the BZ-X800 series software. The video represents ~~the~~ baseline rate of mechanical wound closure and is representative of three identical experiments conducted under the same experimental conditions **(Supplementary Video S1a, b, c)**.

**Supplementary Video S2: Representative wound healing assay of quercetin treated A549 cells.**

Time-lapse phase contrast microscopy showing the migration of A549 cells in the quercetin treated group (50 µM) over a period of 20 hours. Images in the selected region were captured every 20 minutes using Keyence BZ-X800 series microscope. The time lapse images were converted into videos (960 x 720 pixel) using the BZ-X800 series software. The video illustrates the effect of quercetin on the mobility of A549 cells and is representative of three identical experiments conducted under the same experimental conditions **(Supplementary Video S2a, b, c)**.

**Supplementary Video S3: Representative wound healing assay of H520 control cells.**

Time-lapse phase contrast microscopy showing the migration of H520 cells in the control group over a period of 20 hours. Images in the selected region were captured every 20 minutes using Keyence BZ-X800 series microscope. The time lapse images were converted into videos (960 x 720 pixel) using the BZ-X800 series software. The video represents the baseline rate of mechanical wound closure and is representative of three identical experiments conducted under the same experimental conditions **(Supplementary Video S3a, b, c)**.

**Supplementary Video S4: Representative wound healing assay of quercetin treated H520 cells.**

Time-lapse phase contrast microscopy showing the migration of H520 cells in the quercetin treated group (50 µM) over a period a 20hrs. Images in the selected region were captured every 20 minutes using Keyence BZ-X800 series microscope. The time lapse images were converted into videos (960 x 720 pixel) using the BZ-X800 series software. The video illustrates the effect of quercetin on the mobility of H520 cells and is representative of three identical experiments conducted under the same experimental conditions **(Supplementary Video S4a, b, c)**.
